# Design and simulation of an interactive human robot disassembly system for end of life phones

**DOI:** 10.3389/frobt.2026.1885382

**Published:** 2026-10-27

**Authors:** Soufiane Ameur, Mohamed Tabaa, Kaouter Karboub, Mohamed Hamlich, Richard Bearee

**Affiliations:** 1 CCPS Laboratory, ENSAM-C, Hassan II University, Casablanca, Morocco; 2 Pluridisciplinary Laboratory of Research and Innovation (LPRI), EMSI Casablanca, Casablanca, Morocco; 3 LISPEN, Arts et Métiers Institute of Technology, Lille, France

**Keywords:** process design, E-waste, human robot collaboration, human robot interaction, phone disassembly

## Abstract

**Introduction:**

With the growing challenge of e-waste and end-of-life phones, a clear demand is required for efficient and scalable recycling solutions. This study proposes a simulation-based and proof-of-concept framework for a collaborative robotic disassembly system that consists of six specialized stations designed to support the systematic extraction and routing of the main end-of-life phone components. The main contribution is the joint integration of a flexible architecture, more than 20 operational and collaborative constraints, and multimodal voice–gesture interaction for dynamic task reassignment and error recovery.

**Methods:**

This study integrates human-robot collaboration (HRC) and human-robot interaction (HRI) using voice and gesture commands to enable adaptive task reassignment and real-time error correction with reduced downtime. This framework combines simulation-based evaluation, RoboDK modeling, constraint-driven simulation, and HRC scenarios to assess system responsiveness. Over 30 operational and collaborative constraints were mathematically formulated.

**Results:**

The Constraint-Driven Python simulation produced an average cumulative processing time of 955.37 s for a modeled batch of 10 phones over 200 iterations, corresponding to an average simulated processing time of 95.54 s per phone, with error rates ranging from 3.3% to 4.1%. The hand-sign recognition module based on YOLOv12n accurately detected gestures with confidence scores in the range of 0.91–0.94, thus improving safety and non-verbal communication in HRC centers. The execution of voice commands for actions such as “start” and “you take” showed inference times of 2.3–2.8 s, thus validating the feasibility of real-time HRI with two methods. With scenarios such as a human abandoning a task, a robot misclassifying a device, or switching tasks based on screen conditions, HRC reduced downtime and improved task continuity by enabling robots to autonomously handle failed or delayed human operations.

**Discussion:**

A brief discussion highlighted our solution in comparison with rigid systems such as Apple’s. The proposed architecture offers greater flexibility when working on variant inputs, combining robotic precision with human adaptability. The results demonstrate the technical feasibility of combining constraint-driven simulation and multimodal HRI for flexible end-of-life phone disassembly, while full industrial and economic validation remains necessary.

## 1 Introduction

Electronic waste (e-waste) has become the fastest-growing solid waste stream in the world, increasing five times faster than documented recycling efforts. In 2022 alone, the world produced more than 62 million tons’ worth of discarded electronic products and projections indicate an estimated 82 million tons per year by 2030. End-of-life smartphones contribute significantly to this where the WEEE Forum estimates that 5.3 billion devices are expected to be discarded by 2022 (ITU, 2024). Many of them stored in drawers or landfill sites are trapping essential materials such as gold, cobalt, and rare earths, on top of releasing hazardous additives into soil and water (Vuppaladadiyam et al., 2024). In addition to the loss of resources and pollution we also mention that poorly managed telephone waste also exacerbates carbon emissions and health risks associated with the informal sector that underline the urgent need for scalable recovery solutions. To solve this challenge, closed-loop recycling strategies have emerged involving efficient disassembly in which equipment is separated into homogeneous fractions before being shredded or remanufactured. Manual disassembly is still dominant but still suffers from high labor costs and safety risks alongside throughput limitations (Alonso Movilla et al., 2016). While automated shredding avoids manual exposure, it does sacrifice value by mixing heterogeneous materials and eliminating reusable parts. As a result, selective disassembly has emerged as the key pre-treatment step that maximizes value recovery while minimizing environmental impact. The use of robotic systems with selective disassembly is proving that it can be a promising solution that offers repeatability and speed combined with precision when we need to extract valuable components like batteries, screens, or printed circuit boards (Li et al., 2018). Fully automated systems, such as the high-speed cellphone disassembly approach of Figueiredo (Figueiredo, 2018) and industrial systems such as Liam and Daisy (Dassault Systèmes, 2025; Heffernan, 2024), achieve higher throughput and repeatability but depend on dedicated equipment and are generally limited to specific phone models. Optimization-based methods improve disassembly sequencing and human–robot task allocation by considering time, cost, precedence, safety, and ergonomic factors (Lou et al., 2024a), (Chu and Chen, 2023; Lou et al., 2024b; Huang et al., 2021; Shen et al., 2023; Liao et al., 2023). More recent approaches integrate reinforcement learning, Bayesian networks, and digital twins to adapt task allocation under uncertainty. However, most of these studies focus on batteries or predefined products, while the combined integration of mixed-model phone disassembly, multimodal HRI, operational constraints, and dynamic error correction remains limited. The previous generations involved a combination of customized programs and industrial robots and were only viable for uniform product lines. Consequently, robotic disassembly systems are frequently challenged by factors such as product design variability, unexpected defects, and the sudden deformations frequently found in returned or damaged devices (Foo et al., 2022). At this point, human-robot collaboration (HRC) and human-robot interaction (HRI) are becoming vital (Lee et al., 2024). Offering flexible and intuitive cooperation between humans and robots and enabling the disassembly line to be more resilient and responsive than ever before. This study presents a design and simulation of a real-world collaborative disassembly process for EoL cell phones. This compromises a sequence of dedicated stations, where each has been responsible for a specialized task such as sorting, battery extraction, screen removal, or camera extraction, with the aim of achieving 100% component recovery. The article explores the architectural design of each station, the integration of robotic agents and collaboration scenarios, as well as a multi-level simulation and proof-of-concept validation. System feasibility and efficiency are rigorously assessed through a multi-level validation process involving RoboDK simulations, Constraint-Driven Python Simulation models and human-in-the-loop collaboration tests. The main abbreviations used throughout this study are summarized in Table 1.

**TABLE 1:** Abbreviation table.

Abbreviation	Name
HRC	Human Robot Collaboration
HRI	Human Robot Interaction
E-Waste	Electronic Waste
EoL	End of Life
DSB	Disassembly Sequence Balancing
CV	Computer Vision

The remainder of this paper is organized as follows. Section 2 reviews recent literature on robotic disassembly, collaborative robots and intelligent e-waste processing with focusing on current limitations and research gaps. Section 3 outlines the problem formulation and presents the methodological framework that includes the description of each disassembly station and HRI training. Section 4 provides details of the simulation environment and validation process encompassing RoboDK modeling, Constraint-Driven Simulation and a real-life collaborative simulation incorporating HRC scenarios. A comparative analysis is also carried out to benchmark the system’s performance against existing disassembly approaches. And Section 5 concludes the paper by summarizing the main results and discussing implementation prospects outlining potential directions for future research and industrial deployment.

## 2 Related research

Recent research has highlighted advanced methodologies such as the ergonomic task allocation between humans and robots together with dynamic sequencing and adaptive human-robot interactions to further optimize these collaborative systems. As an example, scheduling models that incorporate an ergonomic assessment have demonstrated significant improvements in operator safety and efficiency (Asif et al., 2024). While remanufacturing industries are moving towards Industry 5.0, HRC is playing a major role in building sustainable and intelligent remanufacturing systems adapted to mass customization and the increasing demand for circular economy practices. Hjorth and Chrysostomou (2022) underlined the importance of integrating robots for repetitive tasks and humans for decision-making in non-destructive processes. Lee et al. (2023) presented a comprehensive review of HRC applications with a focus on task allocation frameworks for dynamic recycling scenarios. A high-speed robotic disassembly system is developed for cell phone recycling by Figueiredo (2018), achieving a 95% success rate for non-destructive component recovery. Another example by Lou et al. (2024a) introduces an HCPS-based approach to sequential disassembly planning that relies on a hybrid gray wolf optimization algorithm to improve operator safety along with task efficiency. Xiao et al. (2023) have examined dynamic recycling strategies for electric vehicle batteries in which the challenges of managing battery design variability and the need for adaptive HRC systems are highlighted. Li et al. (2024) explored the use of HRC and AI technologies with a focus on improving task adaptability and recovery rates. In the same vein, Chu and Chen (2023) recently suggested a hybrid particle swarm optimization system that uses Q-learning to optimize task allocation for power battery disassembly and satisfy priority constraints. A customized model for disassembly sequence planning is introduced by Lou et al. (2024b) that incorporated ergonomic assessments as well as a hybrid ant-based optimization algorithm to adapt to specific operator requirements. In another study, Huang et al. (2021) have developed a workflow and sequence planning for EoL products using the bee algorithm, which has been validated by case studies of electric motors as well as battery packs. Shen et al. (2023) discussed HRC disassembly line balancing with the aid of a multi-objective optimization model to improve workstation efficiency and minimize energy consumption. Liao et al. (2023) have built a robust framework providing for the planning of disassembly sequences in the event of uncertainty with the emphasis on cost-effective task allocation between humans and robots. A lithium-ion battery disassembly system based on a digital twin has been suggested by Qu et al. (2023) as a way of improving the flexibility and efficiency of adaptive task sequencing. Further studies have extended these research results through a service platform integrating robotic disassembly scheduling with industrial robots with the aim of improving operational efficiency, for example, the work by Liu et al. (2020). Tan et al. (2021) were able to do a hybrid disassembly framework specifically addressing the disassembly efficiency of electric vehicle batteries by optimizing parameters such as design, safety, and cost. Hellmuth et al. (2021) have assessed a possibility of automating the disassembly of electric vehicle batteries and demonstrated that it is possible to automate repetitive unscrewing tasks while suggesting that lifting tasks be retained by humans. Gao et al. (2024) have employed observable deep reinforcement learning to optimize multi-agent strategies for HRC disassembly of electric vehicle batteries by significantly improving task sequencing and collaboration efficiency. Ionescu et al. (2022) made a propose of a flexible manufacturing technology integrating an autonomous robotic system featuring advanced control and synchronization mechanisms in disassembly and repair operations. Studies have extended HRC disassembly toward more adaptive and human-centered planning. Zhang et al. (2025) applied a Stackelberg game and multi-agent deep reinforcement learning to optimize task allocation in retired-battery disassembly, while Xia et al. combined large language models with Bayesian networks to improve the reliability of collaborative disassembly sequence planning. Xiao et al. (2025) integrated multi-agent reinforcement learning with a multi-scenario digital-twin platform for dynamic sequencing, resource allocation, and physical validation under different operating modes. Human factors have also received greater attention. Li et al. (2019) incorporated accumulated human fatigue into collaborative sequence planning and task assignment. These works demonstrate progress in learning-based allocation, digital-twin validation, and ergonomic assessment, while their joint integration with multimodal HRI and mixed-model phone disassembly remains limited. A summary of the main related research and its contributions is presented in Table 2.

**TABLE 2:** Summary of related research.

Authors	Summary
Hjorth and Chrysostomou (2022)	Review of HRC in industrial disassembly, emphasizing safety and human-robot integration.
Lee et al. (2023)	Framework for task allocation in HRC, focusing on dynamic recycling scenarios.
Figueiredo (2018)	High-speed robotic system achieving 95% success in component recovery during cellphone disassembly.
Lou et al. (2024a)	HCPS-enabled sequential disassembly planning using grey wolf optimization for improved safety.
Xiao et al. (2023)	Dynamic recycling strategies for EoL vehicle batteries with HRC addressing design variability.
Li et al. (2024)	AI-enhanced HRC systems for lithium-ion battery disassembly, improving adaptability and recovery rates.
Chu and Chen (2023)	Hybrid particle swarm optimization with Q-learning for power battery disassembly.
Lou et al. (2024b)	Personalized disassembly sequence planning using ergonomic assessments and hybrid ant lion optimization.
Huang et al. (2021)	Task allocation and sequence planning for EoL products validated in motor and battery case studies.
Shen et al. (2023)	Multi-objective optimization model for HRC disassembly line balancing to minimize energy consumption.
Liao et al. (2023)	Disassembly sequence planning under uncertainty for cost-effective human-robot task allocation.
Qu et al. (2023)	Digital twin-enabled HRC disassembly system for flexible and adaptive lithium-ion battery recycling.
Liu et al. (2020)	Integration of robotic disassembly planning with physical robots
Tan et al. (2021)	Reduction of operational steps, improved disassembly efficiency
Hellmuth et al. (2021)	Developed scoring model for assessing automation potential
Gao et al. (2024)	Optimization of task allocation and sequencing in disassembly
Ionescu et al. (2022)	Enhanced accuracy and flexibility via visual servo assisted ARS
Xia et al. (2025)	LLM-enhanced Dirichlet Bayesian network for reliable HRC disassembly sequence planning under uncertainty.
Zhang et al. (2025)	Stackelberg-game and multi-agent deep reinforcement learning for HRC task allocation and disassembly planning of retired power batteries.
Xiao et al. (2025)	Digital-twin-assisted multi-agent deep reinforcement learning for dynamic HRC task allocation and disassembly-sequence planning of EV batteries.
Li et al. (2019)	Fatigue-aware HRC task allocation and sequence planning using a discrete Bees algorithm for batch-product disassembly.
Liao et al. (2023)	HRC task allocation and sequence optimization using disassembly cost, disassembleability, and quantitative Strain Index assessment.

## 3 Problem statement and methodology

### 3.1 Problem description

Our aim is to develop a dynamic processing chain able to handle end-of-life phones by extracting their components with minimal waste production. What’s more, the main challenge is to design a system capable of adapting to the different configurations and conditions of end-of-life devices while ensuring that each critical component is correctly routed for recycling. To achieve this, the disassembly process must be both flexible, to accommodate structural differences and recycling constraints. Furthermore, the system must enable seamless coordination between disassembly tasks, minimize material losses, and ensure accurate tracking of components throughout the process. To do this, the chain will contain different stations, each of which will extract a single component (Table 3).

**TABLE 3:** Description of each station.

Station	Actor	Collaboration type	Output
EOL Cell Phone Station S0	Human operators, Robots	Synchronized	Sorted EOL cell phones forwarded to the next station; non-desirable items sent to recycling conveyors.
Shell Extraction S1	Human operators	—	Shells for recycling; remaining components forwarded to the next station.
Battery Extraction S2	Robots	Cell	Batteries for safe disposal or recycling; phones forwarded to the next station.
Screen Extraction S3	Human operators, Robots	Coexistence	Intact screens for reuse; damaged screens for recycling; phones forwarded to the next station.
Camera Extraction S4	Robots	Cell	Cameras for recycling; phones forwarded to the next station.
Motherboard Extraction S5	Human operators, Robots	Collaboration	Motherboards for recycling; remaining parts sent to the final recycling station for processing.

#### 3.1.1 Disassembly sequence balancing (DSB)

The disassembly process chain offers 120 possible station sequences as each of the five stations (S1, S2, S3, S4, S5) can be executed in different orders:

$$P\left(S\right)=5!=\left[\begin{array}{c} S1,S2,S3,S4,S5\\ S1,S2,S3,S5,S4\\ \begin{array}{c} S1,S2,S4,S3,S5\\ ... \end{array}\\ \left(120\text{ total sequences }\right) \end{array}\right]$$

However, due to the nature of EOL cell phone designs and the dependencies between components, the process can only begin with either the Shell Removal or Screen Extraction stations while the battery extraction is a second or a third task. This constraint ensures that the outer structure of the phone is addressed first, enabling subsequent stations to access internal components more effectively. By reducing the starting options to these two stations, the system simplifies the workflow while maintaining flexibility in the sequence of the the remaining tasks. The possible logical disassembly sequences are presented in Table 4. Under the constraints that the first station is either S1 or S3 and that S2 occupies the second or third position, the feasible number is:

$$N=2\left(3!\right)+2\left(3\right)\left(2!\right)=12+12=24.$$

**TABLE 4:** Logical sequence possibilities.

S2 is second		S2 is third	
S1 is first	S3 is first	S1 is first	S3 is first
S1 → S2 → S3 → S4 → S5	S3 → S2 → S1 → S4 → S5	S1 → S3 → S2 → S4 → S5	S3 → S1 → S2 → S4 → S5
S1 → S2 → S3 → S5 → S4	S3 → S2 → S1 → S5 → S4	S1 → S3 → S2 → S5 → S4	S3 → S1 → S2 → S5 → S4
S1 → S2 → S4 → S3 → S5	S3 → S2 → S4 → S1 → S5	S1 → S4 → S2 → S3 → S5	S3 → S4 → S2 → S1 → S5
S1 → S2 → S4 → S5 → S3	S3 → S2 → S4 → S5 → S1	S1 → S4 → S2 → S5 → S3	S3 → S4 → S2 → S5 → S1
S1 → S2 → S5 → S3 → S4	S3 → S2 → S5 → S1 → S4	S1 → S5 → S2 → S3 → S4	S3 → S5 → S2 → S1 → S4
S1 → S2 → S5 → S4 → S3	S3 → S2 → S5 → S4 → S1	S1 → S5 → S2 → S4 → S3	S3 → S5 → S2 → S4 → S1

Yet most smartphones require extraction of the shell as the first step in disassembly with some exceptions including iPhones and certain models from certain brands. Such exceptions often require screen extraction first because of the unique construction where the internal components are accessible from the front screen. Several phones are designed with modular back panels which allow the shell to be removed more easily when accessing components such as the battery, motherboard or other parts. Removing the shell is therefore the most practical and widespread approach that will minimize the risk of damaging delicate parts such as the screen and streamlining the disassembly process (show Figure 1).

**FIGURE 1:**
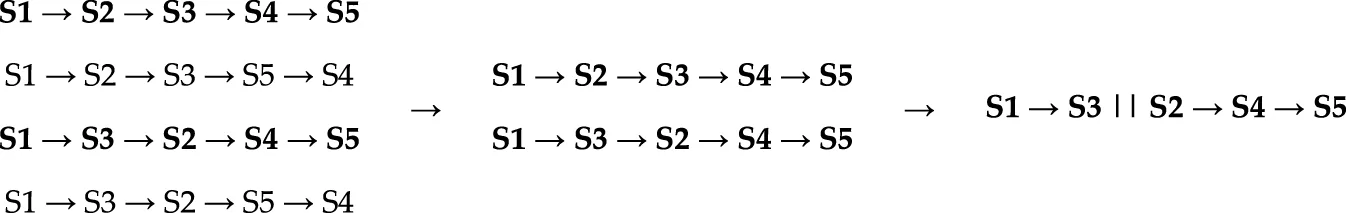
The set of sequences.

Subsequently, the sequences start with S1 (shell removal) is essential for accessing internal components. Then S2 (battery removal) and S3 (screen removal) alternate in priority since the battery and screen must be dealt with early to avoid obstruction during subsequent tasks. Reasons for placing S4 (camera removal) before S5 (motherboard removal) include component accessibility and safety. Since the camera is smaller, fragile and easier to extract it first where paving the way for the motherboard which is usually fixed and more deeply buried. Extracting the camera earlier reduces the risk of damage when disassembling the larger, heavier motherboard. This order ensures a logical workflow from the least to the most complex components, optimizing efficiency and minimizing handling risks.

In this illustrated process, we concluded that S2 (Battery Extraction) and S3 (Screen Extraction) are performed sequentially for the same phone while operating in parallel only on different phones with their order determined by the device architecture and component accessibility. After S1 (Shell Removal) is completed, both the battery and the screen become independently accessible, allowing their extraction to proceed in parallel without dependencies on each other. This parallel execution minimizes idle time for resources and ensures efficient utilization of both human and robotic systems (Figure 2).

**FIGURE 2:**
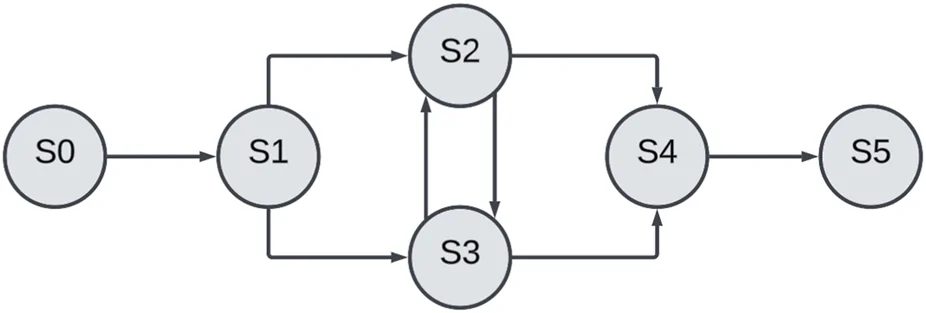
Optimal station sequence.

### 3.2 Proposed station

After finding the minimum sequence between stations, the objective now is to deeply describe the processing chain that will contain the connected stations where each is dedicated to extracting a particular component from the EOL phone where the aim is to produce zero waste. This is achieved by giving each station their own recycling path ( ${\text{RECYCLING}}_{n}$ ) connected by conveyors ${RL}_{n}$ , as shown in Figure 3. The stations can either be operated by robots $R_{n}$ or by human operators $H_{n}$ , or even both working in collaboration. These stations are linked by conveyors $L_{ps,ns}$ that transfer the output of the previous station to the input of the next, all the way to the final recycling station. Each station is equipped with automated conveyors to transport the phones. RFID tagging was made for tracking and diverters directing components to appropriate paths in some stations. This recycling chain is designed to adapt dynamically to the varying conditions of EOL phones (Figure 3).

**FIGURE 3:**
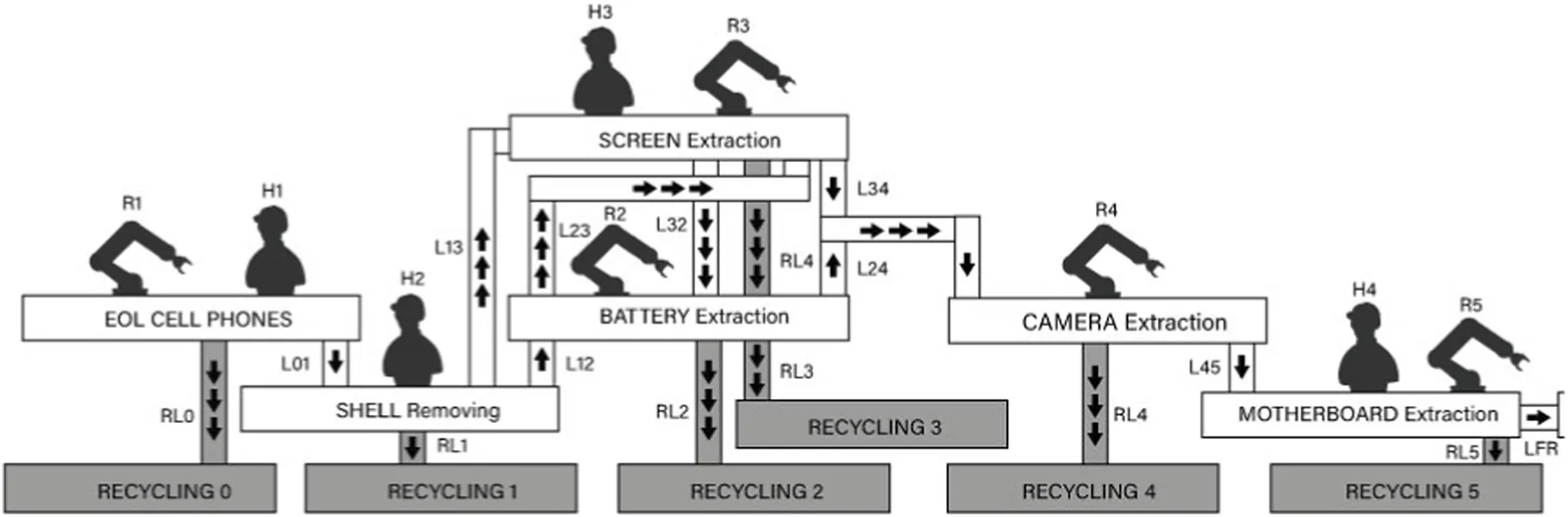
Proposed EOL cell phone disassembly process.

A workflow for the initial disassembly process described in Code 1 provides a structured procedure for detecting and extracting or sorting components according to their condition and type of disassembly. This process contains only an introductory process that summarizes the idea for quicker comprehension. System constraints such as robot accessibility, payload limits or synchronization between conveyors and robotic actions, etc., are not explicitly covered yet. These constraints are essential to guarantee safety and successful operations. Beyond the scope of this initial description, each station will be described in detail in the following sections along with a full breakdown of all constraints.

***Input:*** $I_{n}$

***Initialize:*** $R_{i},L_{ij},RL_{i},d_{i}$ *, error_flag = 0, detection_success*
***For*** *each* $I_{n}$ *:*
 ***If*** $\left(I_{n}\neq CP\right):\left\{R_{1}\to RL_{0}\right\}$

 *error_flag* = 0
 *“detection_success = camera detection accuracy”*
 ***If*** *(detection_success = 0):* $\left\{H_{1}\to RL_{0}\right\}$

 
$L_{01}$

 ***If*** *shell* =1∧ *disassembly_type* = *“rear-type”:*
  
$d_{1}=1,H_{2}\to RL_{1}$

  
$R_{2}\to RL_{2},L_{23}$

  *If (screen_clean* =1 *):* $\left\{H_{3}\to RL_{3}\right\}$

 ***Else:*** $\left\{R_{3}\to RL_{3}\right\}$

 
$L_{34}$

 ***If*** *(screen_clean* =1 *):* $\left\{d_{2}=1,H_{3}\to RL_{3}\right\}$

 ***Else:*** $\left\{d_{2}=0,R_{3}\to RL_{3}\right\}$

 
$R_{2}\to RL_{2},L_{32}$

 
$R_{4}\to$ *camera_search:*
 ***If*** *(camera_visible* =0 *): {Flip-over}*
 ***If*** *(camera_visible* =1 *):* $\left\{R_{4}\to RL_{4}\right\}$

 
$L_{45}$

 
$H_{4}\to$ *unscrew,* $R_{5}\to RL_{5}$

 
LFR

***Output:*** *Disassembled CP*

Code 1 Suggested disassembly process.

The current market offers a wide range of options when it comes to choosing a collaborative robot including KUKA (KUKA, 2026), ABB (ABB Ltd, 2026), UR (Universal Robots A/S, 2026) and others. For our case, we found the UFactory Lite 6 to be an appropriate choice for the proposed EoL phone disassembly line due to its space-saving design and collaborative features (UFactory, 2023). Featuring a 440 mm reach and a 600 g load capacity making it well suited to handling lightweight components such as PCBs or screens in the confined space of a disassembly workstation. Lite 6 enables efficient disassembly cycles while its brushless DC motors ensure smooth and reliable movements. Its open-source control and Python compatibility facilitate seamless integration with simulation tools and the programming of customized tasks. All in all, UFactory Lite 6 offers an accessible and flexible platform for the disassembly of end-of-life electronics, balancing safety, precision and programmability in a compact format (Figure 4).

**FIGURE 4:**
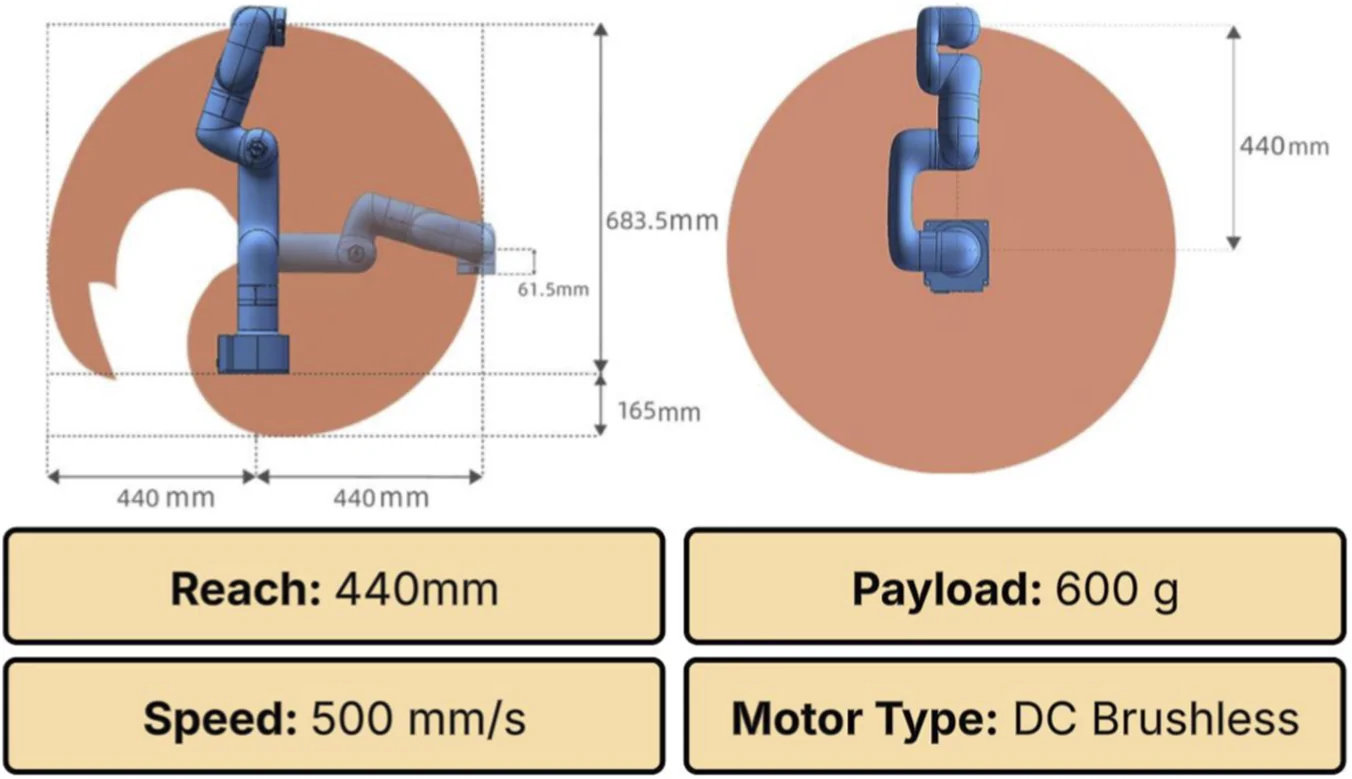
UFactory Lite 6 capabilities.

The architecture of the UFactory Lite 6 collaborative robotic system used in the disassembly station is illustrated in Figure 5. It features a total of six degrees of freedom with each joint offering a specific range of motion. This allows great flexibility and reach in confined workspaces. The cobot can be equipped with two interchangeable end-effectors starting with a conventional gripper and a vacuum gripper. The choice is selected according to the nature of the disassembly task. A Logitech C925e camera is used for real-time object detection and localization (Logitech, 2025). For programming and data visualization, we used the main software UF Studio (UFactory, 2025), which acts also as control interface, translating the camera data into executable commands for the robot. This architecture supports autonomous operation by enabling the cobot to identify and interact with disassembly components dynamically.

**FIGURE 5:**
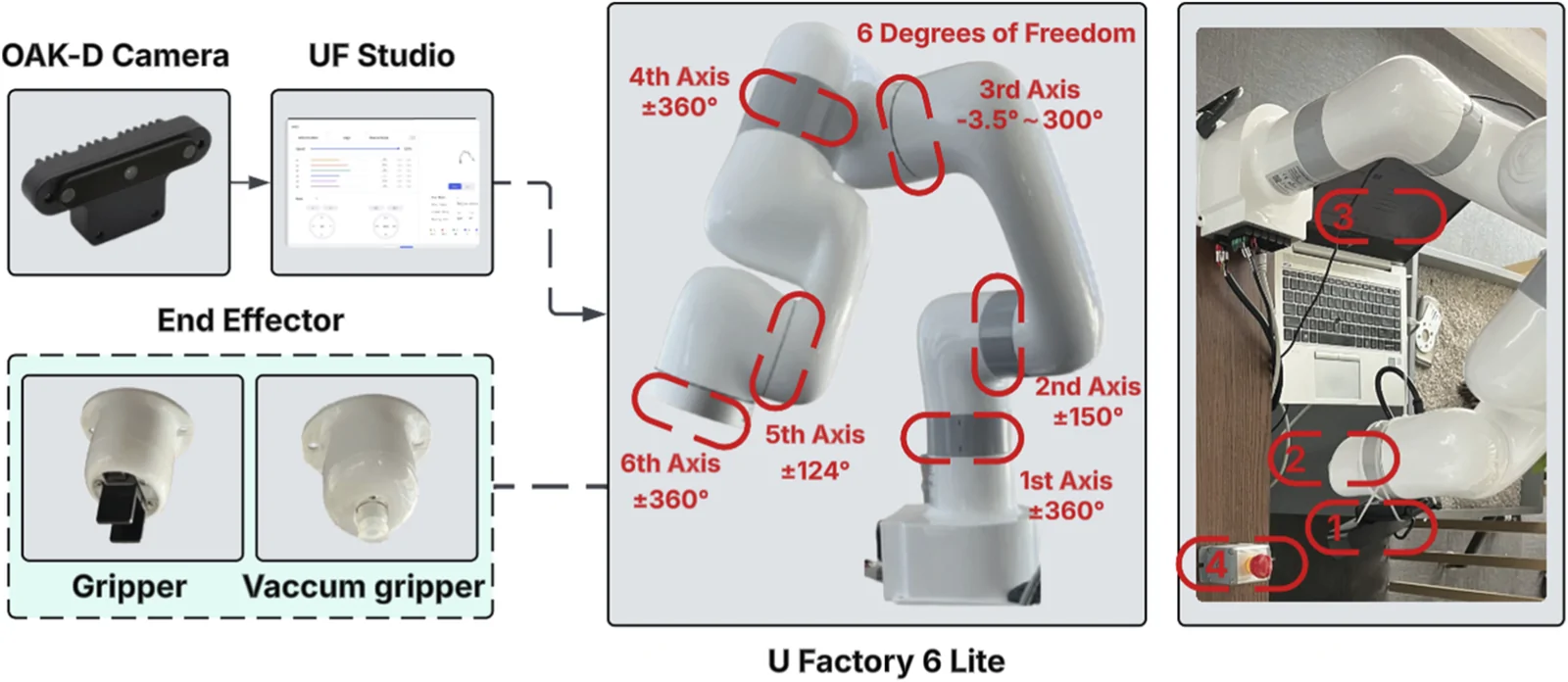
Architecture and end effectors of the UF6 lite.

#### 3.2.1 Objective function

We define the disassembly process of end-of-life mobile phones across five stations: shell removal, screen extraction, battery extraction, camera extraction, and motherboard extraction. Let $N=\left\{1,\ldots ,5\right\}$ denote the set of stations and $G=\left\{R,H,C\right\}$ the set of actors, where R , H , and C represent the robot, human operator, and camera system, respectively. For each station n∈N , $G_{n}\subseteq G$ denotes the actors involved at that station. The station performance function combines the normalized execution time and error rate. The coefficients $\alpha_{n\;}$ and $\beta_{n\;}$ represent the relative importance of time and error at station n , respectively, and satisfy $\alpha_{n}+\beta_{n}=1$ . The global performance function is obtained by summing the individual performance measures of all five stations, thereby providing an overall evaluation of time and error across the complete disassembly process. Mathematically, the global function is expressed as follows:

$$F=\sum_{n=1}^{5}\;\left(\alpha_{n}\sum_{g\in G_{n}}\;T_{n,g}+\beta_{n}\sum_{g\in G_{n}}\;E_{n,g}\right)$$ (1)

$$\alpha_{n}+\beta_{n}=1$$ (2)

The objective function (Equations 1, 2) is used as a performance index to evaluate processing time and error rather than as a formally solved LP/MILP optimization problem.

#### 3.2.2 Station 0: EOL cell phones

Ideally, this station is located at the beginning of the process when cell phones are brought in. However, there is always the possibility that this input contains unwanted waste other than cell phones. For this purpose, there’s an $R_{0}$ robot positioned to detect whether the input is desired or unwanted. In the case of an unwanted item, the robot picks it up and deposits it in the recycling conveyor for alternative processing. If the product is required (EOL Cell Phone), the robot allows it to pass onto the conveyor belt. Using the camera connected to the robot which can make the decision and give the order using object detection technology. The aim is to optimize the station’s processing time Ta∈R∣0≤Ta≤N as well as the error rate Ea∈R∣0≤Ea≤1 . While calculating the time required for this process Ta, many parameters must be taken into consideration like the time required for camera processing tc. There are multiple environmental and operational parameters $\left\{I,D,L,R\in R\right\}$ expressed in seconds that influence the processing time to detect an object and send a command. I is the image processing complexity, D the dust level, L the lighting conditions and R the resolution. where $k_{0}$ is a constant term, and $k_{1},k_{2},k_{3},k_{4}$ , are coefficients expressed in seconds and quantify the additional processing time associated with each operating condition. They are calibrated from experimentally measured camera-processing times using a least-squares regression procedure. The time required for the robot to complete the task ${tr}_{i}$ is calculated as the total time required for the robot to perform its tasks. This includes approaching the object, grasping it or placing it at its destination and returning to its starting position. The time generally is influenced by factors such as the distances involved, the robot’s speed or the gripper actuation time. As shown in Equations 3, 4.

$$0.001\sec<tc=k_{0}+k_{1}.I+k_{2}.D+k_{3}.L+k_{4}.R<3\sec$$ (3)

$${tr}_{i}=tapproach_{i}{+tgrasp}_{i}+tplace_{i}+treturn_{i}$$

$$tapproach=\frac{d}{v_{r}}\;tplace=\frac{d_{place}}{v_{r}}\;treturn=\frac{d_{return}}{v_{r}}$$ (4)

xa is a binary variable which takes value 1 if the human corrects an error committed by the robot, and the value 0 otherwise. Speaking of the human, th represents the time required to complete the human operator’s task, whereas sa indicates the switching time between the robot and the human operator (Equation 5).

$$Ta=\sum_{i\epsilon N}{tc}_{i}+{tr}_{i}+{xa}_{i}\left({th}_{i}+{sa}_{i}\right)$$ (5)

On the other hand, we must bear in mind that when using this technology, there is always a risk of error Ea given that the input involves a high degree of uncertainty, and it is extremely difficult to obtain a 100% prediction. In this case, we suppose that the human operator $H_{1}$ cannot make errors eh=0 and he’s responsible for correcting any errors caused by the robot. The error rate of the robots er $=\left\{x\in R|0\leq x\leq 1\right\}$ is calculated as the number of times the human performs the correction $\sum_{i\epsilon c}{xa}_{i}\;\left(\;C=\left\{1\;.\;.\;.\;N\right\}\;\right)$ divided by the number of entries N . As shown in Equations 6, 7.

Ea=er+eh (6)

$$er=\frac{\sum_{i\epsilon c}{xa}_{i}}{N}$$ (7)

The main operational constraints of Station 0 are summarized in Table 5.

**TABLE 5:** Constraints of station 0.

Constraints
*Binary variable constraint 1*
*Camera and convoyer constraint*
*Distance separating objects*
*Reachability constraint*
*Payload constraint*

#### 3.2.3 Station 1: shell removing

On this station, the input is guaranteed to be an end-of-life cell phone. So, the first objective is to extract the back shell of the phone, something that has been done by human hands, as this requires greater precision. First thing to check is the existence of the shell, also as the type of disassembly, as to remove the shell, it must be a rear-type disassembly. Once the shells have been extracted, they will be sent for recycling, whereas the rest of the phone will follow one of two paths, depending on the type of disassembly. Extracting the shell will simplify the rest of the disassembly process. We use $Gn=\left\{Hc\cup Cv\right\}$ as the main actors with Hc as the human operator and Cv as the conveyor. Concerning the weights of this station $\omega_{b}$ , $\alpha_{b}$ present the weight of Time while $\beta_{b}$ for the error rate. When calculating the time necessary for this station Tb (Equation 8), it is essential to take into consideration the time required for the human th to remove the shell from the phone, in addition to the time required for the conveyor which will send the rest of the phone. There are two conveyors in this case ( ${tc}_{12},{tc}_{13}$ ), of which only one will be operating at a time.

$$Tb=\sum_{i\epsilon N}{th}_{i}+{\left(x{tc}_{12}+{ytc}_{13}\right)}_{i}$$ (8)

However, the time required by the human th (Equation 9) in this process is equivalent to the time taken for the shell to be extracted from the phone te , together with the time needed for the human to determine in which channel the phone will proceed tp . The coefficient k reflects variations in human performance, such as effectiveness and skill level.

$${th}_{i}=\left({xb}_{i}{te}_{i}+{tp}_{i}\right).k_{i}$$ (9)

Where:

$$\left\{\begin{array}{l} k>1\;above\;average\;performance\\ k≅1\;average\;performance\;\\ k<1\;below\;average\;performance \end{array}\right.$$

As for the error rate at this station Eb (Equation 10), this can be described as the decision error of the human eh choosing the path the phone will take. Before we can find out if the human has made an error when deciding the path, we first must find out how the system works. Once this station has been passed, that phone will proceed to either the battery extraction $S_{3}$ or the screen extraction ${\;S}_{4}$ , according to priority. For instance, if the phone is supposed to be directed to the S3 but the operator has placed it in the S4, the phone will be rejected and transferred to the S4 before being redirected to the S3 once again.

$$Eb=eh=\frac{\sum A_{i}}{N}$$ (10)

This repetition will indicate the error that has been made, and to demonstrate this we need to add a counter A (Equation 11) which will track the phone for the number of times it has reached a station. This means, if the phone passes one of the stations more than once, it will be assumed to be an error, where:

$$\left\{\begin{array}{l} A=1\;if\left(S_{4}\left.>1\|\right\|S_{3}>1\right)\\ A=0\;otherwize\; \end{array}\right.$$ (11)

The main operational constraints of Station 1 are summarized in Table 6.

**TABLE 6:** Constraints of station 1.

Constraints
*Binary variable constraint 2*
*Decision time constraint*
*Human performance constraint*

#### 3.2.4 Station 2: battery extraction

The extraction of the battery will be performed only by a robot, given that it involves a high level of risk. To accomplish this, a robot must be equipped with a camera to detect the existence and positioning of the battery. Two inputs are provided in this station, one originating from the previous shell extraction station, where cell phones are sent with a rear-type disassembly, since the battery is mainly extracted from the rear. Then another input coming from the screen extraction station, where the screen is extracted first. Once the robot has detected the existence and location of the removed item, it takes it to the recycling station for subsequent processing. Finally, if the screen is available, the phone will be sent to the screen extraction station through a diverter system pushing the phone towards $L_{23}$ conveyor, otherwise it will be sent directly to the camera extraction station.

When calculating the time required for this station $T_{c}$ (Equation 12), we need to take into consideration the time required for the camera tc to detect the object, followed by the time required for the robot tr for picking up and placing the target, plus the time required for the diverter tdv to switch the phone’s path. Lastly, the time taken for the conveyor $L_{23}$ to send the phone to the screen station tcv .

$$T_{c}=\sum_{i\epsilon N}{tc}_{i}+{tr}_{i}+{xc}_{i}\left({tdv}_{i}+{tcv}_{i}\right)$$ (12)

There are many possible reasons for errors at this station, notably if the camera does not detect the battery correctly or if the robot has the wrong target, and if it malfunctions. Alternatively, an error may be caused by the deviator installed in this station which may not switch the phone correctly or may not work at all. The importance of each error in this station is expressed by the multipliers $k_{0},k_{1},k_{2}\;\left\{k_{n}\in R|0\leq k_{n}\leq 1\right\}$ (Equation 13).

$$E_{c}=e_{c}.k_{0}+e_{r}.k_{1}+e_{dv}.k_{2}$$ (13)

The main operational constraints of Station 2 are summarized in Table 7.

**TABLE 7:** Constraints of station 2.

Constraints
*Diverter variable constraint 3*
*Diverter and conveyor constraint*
*Diverter’s time constraint*
*Distance separating objects*
*Reachability constraint*
*Payload constraint*

#### 3.2.5 Station 3: screen extraction

Unlike the previous station, in which the robot carries out the task itself, this one involves collaboration between human and robot, working in parallel. Humans are responsible for extracting the screens that are still in good condition, while the robot extracts screens that are in worse condition. This station is supplied with two inputs, one from the previous shell extraction station, where cell phones are sent with a front-type disassembly, given that the screen is mainly extracted from the front. A second input comes from the battery extraction station, which is where the battery is extracted first. Both displays, in good or bad condition, are sent to the recycling station for later processing, where batteries are re-verified their existence before being sent to the battery station. If both components are extracted, the rest of the phone is sent to the next station (Figure 6).

**FIGURE 6:**
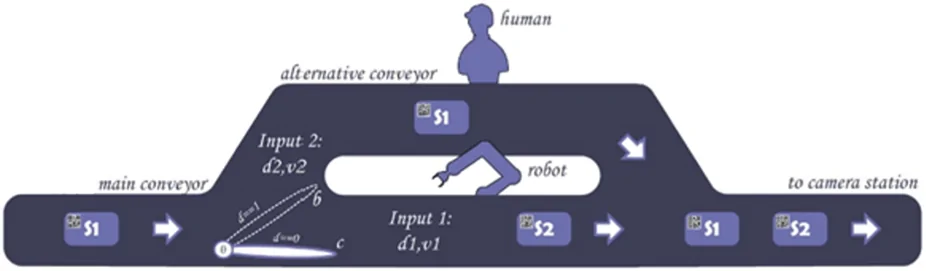
Illustration of station 3.

In determining the time needed for this station ${\;T}_{d}$ (Equation 14), we consider the time needed for the diverter tdv to change the phone’s path in the event of a switch. Finally, the time required for the human or robot to extract the screen from the phone. In which only one of these two (human th , robot tr ) is executed at a time.

$$T_{d}=\sum_{i\epsilon N}x_{i}{tr}_{i}+y_{i}\left({th}_{i}+{tdv}_{i}\right)$$ (14)

Regarding errors in this station ${\;E}_{d}$ (Equation 15), we’re only going to consider the error of a bad decision regarding the disassembly method, which are represented in two cases (Equation 16), a robot has received a clean screen, or a human has received a damaged screen. Both cases contradict the objective of the line. This error may be caused by either the RFID $e_{r}$ or the diverter system $e_{dv}$ , or a combination of both. Calculating this error is based on adding a counter c which increments each time the phone takes a wrong path, Divided by the number of entries. As shown in Equations 6, 7. $I_{1}$ and $I_{2}$ represent the inputs of each channel.

$$E_{d}=e_{r}+e_{dv}=\frac{\sum c_{i}}{I_{1}+I_{2}}$$ (15)

$$\left\{\begin{array}{l} c=1\;if\left(I_{1}\neq S2\;\|\;I_{2}\neq S1\right)\\ c=0\;otherwize\; \end{array}\right.$$ (16)

The main operational constraints of Station 3 are summarized in Table 8.

**TABLE 8:** Constraints of station 3.

Constraints
*Binary variable constraint 4*
*Robot’s time constraint*
*Diverter variable constraint*
*Heater constraint*
*Distance separating objects*
*Reachability constraint*
*Payload constraint*

#### 3.2.6 Station 4: camera extraction

This station is designed to pull the camera out of the cell phone. It is fed by both the screen and battery extraction stations (Input 1, Input 2). Just as the battery extraction station, all tasks are carried out by robots. The phones arriving from the inputs of this station are randomly placed as they can either be placed at their front or reversed to their back, thus making it difficult for the camera to be identified by the robot as it can be hidden. For this purpose, a flip-over mechanism has also been installed to reverse the side of the phone in case the robot fails to detect the camera (Figure 7). Next, after the robot detects the existence and the location of the camera for an automated extraction, the camera is then taken to the recycling plant for further processing. To determine the time needed for this $T_{e}$ station (Equation 17), we consider the time needed for the flipping unit tf to change the side of the phone in the event of flipping. Finally, the time needed for the robot to extract the camera from the phone. In general, the unit timings are controlled by binary variables ( x,y ). The efficiency of the robot and the camera system are also considered, where higher values indicate better performance and reduced errors. Furthermore, the coordination between these units is crucial to ensure smooth and time-efficient operation.

$$T_{e}=\sum_{i\epsilon N}x_{i}{tf}_{i}+y_{i}{tr}_{i}$$ (17)

**FIGURE 7:**
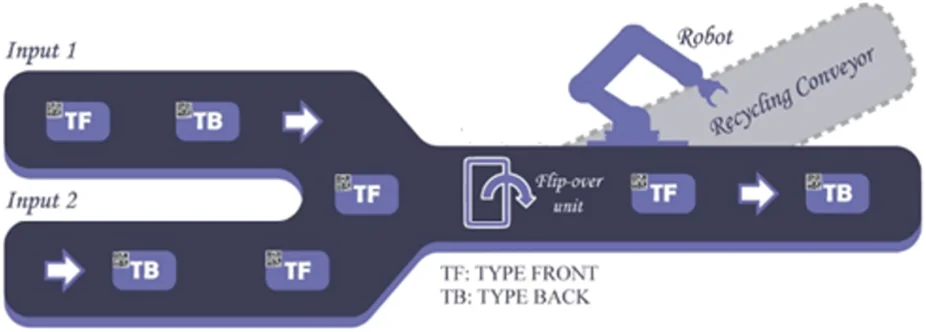
Illustration of station 4.

Errors at this station can be due to a number of reasons such as incorrect detection of the object by the camera, incorrect extraction of the camera by the robot, or system malfunction. It is also possible that the rotation unit used in this station does not rotate the phone properly or does not work at all. The importance of each error in this station is expressed by multipliers $k_{0},k_{1},k_{2}$ . They quantify the impact of each type of error on overall system performance. Ensuring that the performance coefficient k remains high can mitigate these errors effectively. Maintaining a high coefficient of performance is an effective way of mitigating these errors (Equation 18).

$$E_{e}=e_{r}.k_{0}+e_{r}.k_{1}+e_{f}.k_{2}$$ (18)

The main operational constraints of Station 4 are summarized in Table 9.

**TABLE 9:** Constraints of station 4.

Constraints
*Binary variable constraint 5*
*Flip-over unit constraint*
*Station’s inputs constraint*
*Distance separating objects*
*Reachability constraint*
*Payload constraint*

#### 3.2.7 Station 5: motherboard extraction

The station is designed to extract motherboards from old cell phones which involves initial checks and then removal and recycling of the motherboard via specific channels. It works through a human-robot collaboration, with both operators responsible for unscrewing the printed circuit board and extracting the motherboard if identified. The board is then sent to the recycling station (Figure 8). Once the five components or less have been extracted, there are still many more parts to be reconditioned since the aim is to have a 100% recycled cell phone, meaning that a final recycling station is responsible for processing all the remaining parts. For a collaborative approach, the Station Equation needs to evolve to include key factors such as task handovers and shared task execution and probabilistic delays caused by human-robot interactions. Such refinement is necessary to reflect the true complexity and temporal variability of collaborative disassembly. As demonstrated in Equation 19

$$T_{f}=T_{unscrew}+T_{handover}+T_{extract}+E\left[T_{interaction}\right]=\max\;\left(\sum_{i\in H}\;t_{h,i},\sum_{i\in R}\;t_{r,i}\right)+T_{handover}+T_{extract}+\sum_{k=1}^{K}\;P_{k}\cdot \Delta tk$$ (19)

$T_{f}$ was expanded to account for the disassembly process’s granular aspects: unscrewing, task handover, motherboard extraction, and interaction time. The first term represents the sum of unscrewing times $,T_{unscrew}$ , divided between the human and the robot based on $x_{i}$ and $y_{i}$ . The terms $T_{\text{handover}}$ and $T_{\text{extract}}$ represent the measured handover and motherboard-extraction durations. Finally, $E\left[T_{interaction}\right]$ is the expected interaction time, computed as the sum of probabilities where $P_{k\;}$ is the probability of interaction event k and $\Delta t_{k\;}$ is its associated delay. All time-related terms are expressed in seconds. In a process involving the human and the robot on the recycling conveyor system, errors can arise from various sources. Starting from the error during the unscrewing process, such as incorrectly removing screws. The camera error can stem from inaccuracies in detecting and identifying the screw or the motherboard in the phone, which is critical for the robot’s subsequent action (Equation 20).

$$E_{f}=e_{s}+e_{e}$$ (20)

**FIGURE 8:**
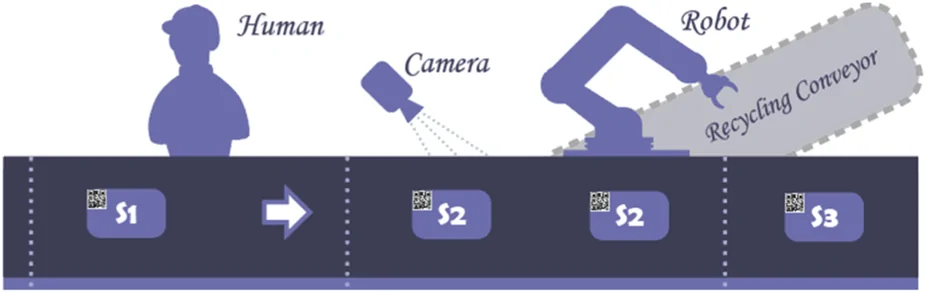
Illustration of station 5.

Errors concerning unscrewing can set up a counter to record every instance where the unscrewing task is carried out incorrectly. This might involve errors such as missing screwdrivers or damaged screws inside future phones. The error is real when the camera has detected a screw in the extraction phase (Equations 21, 22).

$$e_{s}=\frac{\sum s_{i}}{N}$$ (21)

$$\left\{\begin{array}{l} s=1\;if\;S2\;has\;screws\;\\ s=0\;otherwize\; \end{array}\right.$$ (22)

Errors concerning unscrewing can set up a counter r to record every instance where the unscrewing task is carried out incorrectly. This might involve errors such as missing screwdrivers or damaged screws inside future phones. The error is real when the camera has detected a screw in the extraction phase (Equations 23, 24).

$$e_{e}=e_{c}+e_{r}=\frac{\sum r_{i}}{N}$$ (23)

$$\left\{\begin{array}{l} r=1\;if\;S3\;has\;a\;motherboard\;\\ r=0\;otherwize\; \end{array}\right.$$ (24)

The main operational constraints of Station 5 are summarized in Table 10.

**TABLE 10:** Constraints of station 5.

Constraints
*Binary variable constraint 6*
*Unscrewing time constraint*
*Camera time constraint*
*Distance separating objects*
*Reachability constraint*
*Payload constraint*

#### 3.2.8 Constraints

##### 3.2.8.1 Constraint 1: binary variable constraint 1

If the camera has not correctly detected an input In as belonging to a non-EOL cell or has detected a cell phone CP as an unwanted object. In that case, xa takes the value of 1, meaning that the human need to correct the error made by the robot otherwise it takes the value 0 (Figure 9).

$$xa\;\left\{\begin{array}{c} 1\;if\;\left(In\neq CP\right)\\ 0\;if\;othewize \end{array}\right.$$

$$In\neq CP\;if\;\left(D>D_{\max}\right)\vee \left(L<T_{low}\vee L>T_{high}\right)\vee \left(R_{u}=1\right)$$

$$R_{u}=1\text{ if input is untrained},\text{else }R_{u}=0$$ (25)

**FIGURE 9:**
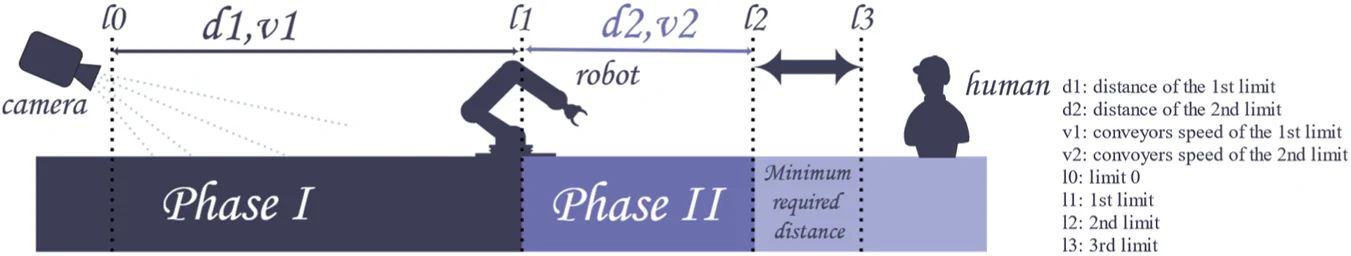
Illustration of constraint 2.

Under difficult operating conditions such as excessive dust levels, low or too-bright lighting or even when the input does not belong to the trained recognition set, it is important for the system to ensure that the type ( $I_{n}$ ) does not match the classification ( $C_{p}$ ). This constraint of mismatch is dynamically activated when specific thresholds are exceeded, such as dust level (D) above $D_{\max},L$ ighting ( L ) outside the acceptable range $\left[D_{low},D_{high}\right]$ or when the input is not recognized $\left(R_{u}=1\right)$ .

##### 3.2.8.2 Constraint 2: camera and conveyor constraint

Before running the conveyor, we need to set a constraint that ensures the conveyor speed is matched or higher than the camera processing time tc , since it may be possible that the time needed for the conveyors to move from point a to point b is not adequate for the camera to detect the object correctly. We considered that the distance between the object and the robot $d_{t}$ must be at least 40 cm. The distance $d_{t}$ will be divided into two phases: phase I and phase II. The first phase is a variant, given that $l_{0}$ the point at which the camera can track cannot be accurately estimated, if the camera still fails to classify the object and the object crosses the threshold $l_{1}$ , it switches to phase II, in which the speed will be slower $v_{1}\geq v_{2}$ allowing longer time for the camera to identify the object. $l_{2}$ represents the threshold for phase II, as exceeding it will go into the minimum distance required for the robot to position itself to pick up the object. In other words, if the camera has detected the object as being undesirable between $l_{2}$ and $l_{3}$ , the robot cannot perform the task correctly. In this instance, humans handle responsibility. As defined in Equations 26–29.

$$d_{t}=d_{1}+d_{2}\geq 40\;cm$$ (26)

$$d_{2}=l_{2}-l_{1}=15\;cm$$ (27)

$$tc\leq \frac{d_{1}}{v_{1}}+\frac{d_{2}}{v_{2}}$$ (28)

$$if\;\left(l_{2}\leq d\;{\leq l}_{3}\;\&\&\;c==0\right)\left\{\begin{array}{c} \;true\;v==v2\;\\ else\;v==v1\; \end{array}\right.$$ (29)

##### 3.2.8.3 Constraint 3: distance separating objects

As mentioned previously, to obtain better robotic sorting, we should have a minimum distance of 40 cm, otherwise if two objects are close to each other, the robot will have trouble grabbing them both (Figure 10). To ensure this, we simply add a barrier b to guarantee this minimum separation. The conveyor instructs the barrier to stop when the required distance is reached (Equation 30).

$$if\;\left(d\geq 40\;\|\;d\geq l_{1}\right)\left\{\begin{array}{c} \;true\;b=0\;\\ else\;b=1\; \end{array}\right.$$ (30)

**FIGURE 10:**
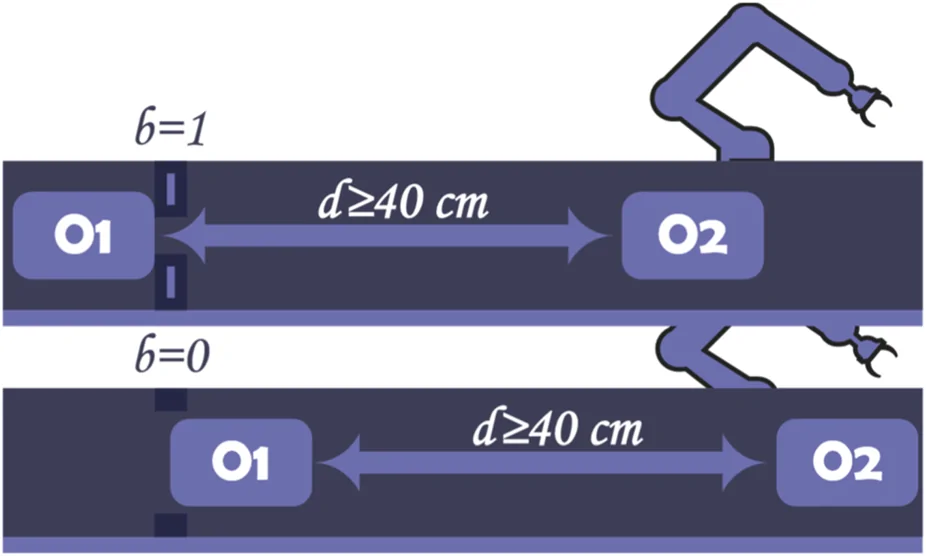
Illustration of constraint 3.

##### 3.2.8.4 Constraint 4: reachability constraint

The robot’s end-effector position is determined using the forward-kinematics model based on the Denavit–Hartenberg parameters. The Cartesian position (Equation 31) is represented by:

$$P\left(\theta \right)=\left[\begin{array}{l} x\left(\theta \right)\\ y\left(\theta \right)\\ z\left(\theta \right) \end{array}\right]$$ (31)

A target position $P_{t}$ Equations 32, 33 is considered reachable if there exists a valid joint configuration satisfying:

$${\left\|P\left(\theta \right)-P_{t}\right\|}_{2}\leq \varepsilon$$ (32)

subject to:

$$\theta_{i}^{\min}\leq \theta_{i}\leq \theta_{i}^{\max},i=1,\ldots ,6$$ (33)

As an initial workspace condition (Equation 34), the target must also remain within the robot’s rated reach:

$${\left\|P_{t}-P_{\text{base}}\right\|}_{2}\leq 440\text{ mm}$$ (34)

To avoid collisions, the robot must maintain a minimum distance from each obstacle (Equation 35):

$${\left\|P\left(\theta \right)-O_{j}\right\|}_{2}\geq D_{\text{safe}}$$ (35)

where $O_{j}$ is the position of obstacle $j,D_{\text{safe}}$ is the minimum safety distance, and ε is the accepted positioning tolerance.

##### 3.2.8.5 Constraint 5: payload constraint

A robot’s load capacity is essential to ensure safe and efficient operation. The torque τ generated at each joint by the payload W (Equation 36), must not exceed the joint’s torque limit τ⁡max⁡,i .

$${\;\tau \;}_{i}=W\cdot g\cdot d_{i}$$

$$\tau i\leq \tau \max,i,\forall i\in \left\{1,2,\ldots ,6\right\}$$

$$W<\;W\left(d_{CoG}\right)$$ (36)

$$W\left(d_{CoG}\right)=\left\{\begin{array}{l} 0.6\;if\;d_{CoG}\leq 350\;mm\;\\ 0.6-{\left(\frac{d_{CoG}-350}{250}\right)}^{2}.\left(0.6-0.35\right)\;if\;{350\;mm\leq d}_{CoG}\leq 600\;mm \end{array}\right.$$

When expressing W ( $d_{CoG}$ ) (payload capacity as a function of center-of-gravity offset), our UF lite 6 payload curve shows a convex transition between 350 mm and 600 mm in which payload initially decreases sharply then flattens out as the center-of-gravity offset approaches 600 mm.

##### 3.2.8.6 Constraint 6: binary variable constraints 2

In terms of the binary variables in this station, the first relates and controls whether the human has performed the extraction of the shell. If xb is set to 1, this means that the human has extracted the shell, if not, it is set to 0 (Equation 37).

$$\left\{\begin{array}{l} xb=1\;if\;there^{\prime }s\;no\;shell\\ xb=0\;if\;othewize\; \end{array}\right.$$ (37)

This second parameter is reserved for the phone path in which the station has two conveyors $\left(c_{12},c_{13}\right)$ presenting the output. In this case, only one of the conveyors can be activated by using two variables x and y .

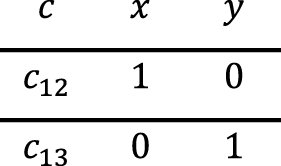




##### 3.2.8.7 Constraint 7: decision time constraint

For tracking purposes, we require a simple way of identifying an end-of-life phone at each station. The most efficient method is to label it with a serial number, as its detection using object detection can be problematic due to the high level of similarity in this use case. In an ideal situation, the serial number should be presented in the form of an RFID tag to facilitate identification and decision-making in future stations reducing reliance on camera identification (show Figure 11). The RFID tag can be repositioned on the phone each time a part is removed to ensure that it remains intact throughout the disassembly process. The time required to place the RFID tag tq, is included in the decision time td , as is the time required to place the phone on the selected conveyor tp (Equation 38). It is important to understand that adding tasks can affect the process so we’ll need to set the overall time to a value lower than the conveyor time.

$${td}_{i}={tq}_{i}+{tp}_{i}\leq \frac{dc}{{vc}_{1}}-{te}_{i}$$ (38)

**FIGURE 11:**
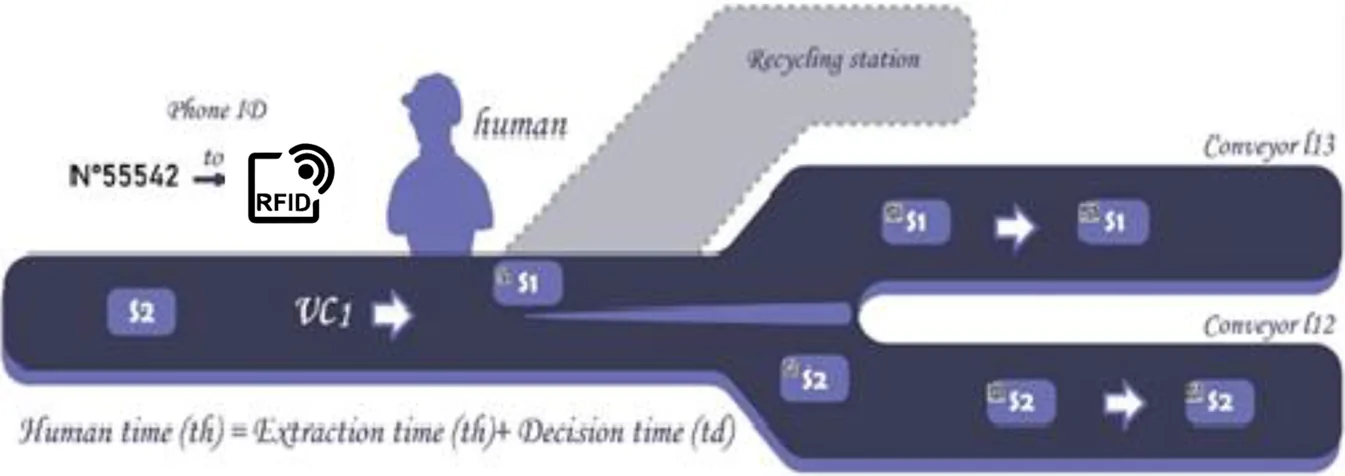
Illustration of constraint 7.

##### 3.2.8.8 Constraint 8: human performance constraint

To demonstrate the relationship between the performance coefficient k and the number of errors A , it is necessary to use Pearson correlation by calculating the Pearson correlation coefficient between these two variables. The Pearson correlation coefficient r (Equation 39) measures the linear relationship between two variables whereby r=1 indicates a perfect positive linear relationship, rp=− 1 indicates a perfect negative linear relationship and r=0 indicates no linear relationship. Where $\bar{\;A},\bar{\;k}$ are the means of A,k.

$$r=\frac{\sum_{i=1}^{N}\left(k_{i}-\bar{k}\right)\left(A_{i}-\bar{A}\right)}{\sqrt{\sum_{i=1}^{N}{\left(k_{i}-\bar{k}\right)}^{2}}\sqrt{\sum_{i=1}^{N}{\left(A_{i}-\bar{A}\right)}^{2}\;}}$$ (39)

In such cases, the human performance coefficient k (Equation 40) must be kept above a minimum threshold which will reduce the probability of errors during the decision task.

$$k\geq k_{\min}$$ (40)

##### 3.2.8.9 Constraint 9: diverter variable constraint

There are two distinct states of operation for activated and deactivated, represented by d=0 and d=1 respectively. If the diverter is deactivated ( d=0 ), it is positioned so that the items on the conveyor continue their main path towards the camera station. When deactivated, the diverter is aligned to allow direct passage and its duration has no effect on system operation. However, when the diverter is activated ( d=1 ) it pivots to redirect items from their main path to another path (filter station). This activation state implies that the diverter arm rotates around a fixed pivot point D. As shown in Equation 41.

$$if\;\left\{\begin{array}{c} \;d=1\;Diverter\;is\;closed.\;\to \left({tdv}_{i}+{tcv}_{i}\right)\neq 0\;\\ d=0\;Diverter\;is\;opened.\;\to \left({tdv}_{i}+{tcv}_{i}\right)=0 \end{array}\right.$$ (41)

##### 3.2.8.10 Constraint 10: diverter and convoyer constraint

Ensuring the smooth operation of the conveyor and diverter system means regulating the speed of both components so that the conveyor does not overtake the diverter. This helps avoid collisions and ensures a smooth transition of items between conveyor and diverter (Figure 12). The conveyor speed *v*1 must always be lower than the diverter speed vb , which is a function of the diverter length l and its angular velocity ω . Point θ is the pivot so its coordinates are fixed. The ends of the diverter arms that pivot around point D and move towards point B (or C) are called point E (Equation 42). Once the diverter rotates its coordinates change. Coordinates of point E ( $E_{x},E_{y}$ ) as a function of angle α are:

$$\left\{\begin{array}{c} E_{x}=\theta_{x}+l\cos\left(\propto \right)\\ E_{y}=\theta_{y}+l\sin\left(\propto \right) \end{array}\right.$$ (42)

**FIGURE 12:**
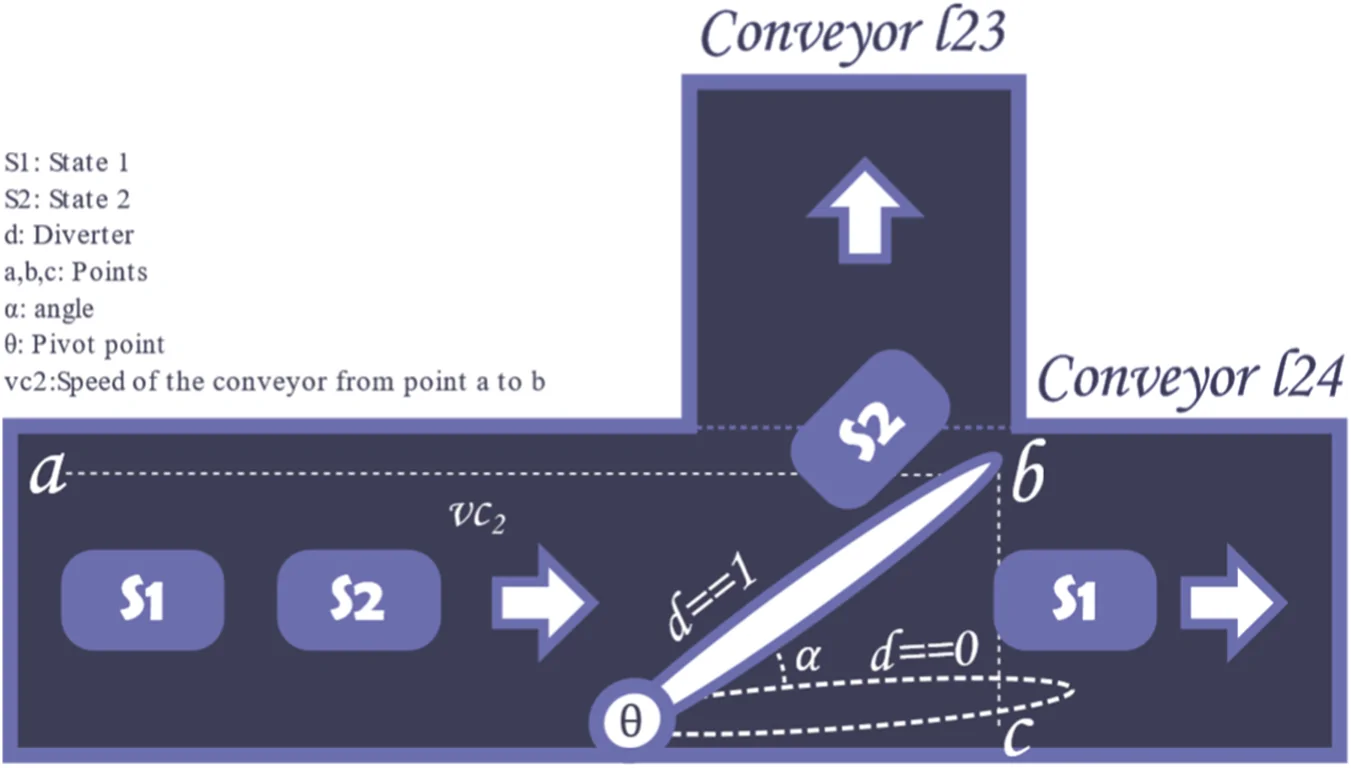
Illustration of constraint 10.

The calculation of the diverter’s linear speed is crucial for ensuring the efficient operation of a conveyor-diverter system. The diverter pivots around a fixed point θ , and its end point E , moves with a linear speed that depends on the angular velocity *ω* and the length of the diverter arm l . we obtain the horizontal and vertical components of the linear speed ${ve}_{x}\;and\;{ve}_{y}$ (Equation 43) of point E. Then, the linear speed ve of point E is then calculated as

$$ve=\sqrt{{\left({ve}_{x}\right)}^{2}+{\left({ve}_{y}\right)}^{2}}$$ (43)

Simplifying:

$$ve=\sqrt{{\left(-l\sin\left(\propto \right).\omega \right)}^{2}+{\left(l\cos\left(\propto \right).\omega \right)}^{2}}$$ (44)

$$ve=\sqrt{l^{2}\omega^{2}\left(\sin^{2}\left(\propto \right)+\cos^{2}\left(\propto \right)\right)}=\sqrt{l^{2}\omega^{2}}=l\omega$$ (45)

Once the speed of the diverter ve (Equations 44–46) has been determined, it is important to avoid collisions and ensure that objects are moved correctly. Introducing a safety margin variable s where $\left\{s\in R|0\leq s\leq 1\right\}$ . Ensuring the diverter speed is kept within safe limits. Finally, the conveyor speed ${vc}_{2}$ should be less than the speed of the diverter at point E. For this, we impose the constraint:

$${vc}_{2}\leq s.ve$$ (46)

##### 3.2.8.11 Constraint 11: diverter’s time constraint

The time needed to move the diverter tdv (Equation 47) needs to be considered carefully with incoming phones, since the process can be affected by inappropriate timing. In the event of a phone being transferred to the screen station S2 , the diverter must remain triggered until the phone is on the next conveyor, considering the upcoming phone. There are two limits to the diverter, the first one $l_{1},$ at the diverter entrance with a second one $l_{2},$ on the inlet of the second conveyor. The speed of the phone moving in the conveyor ${vc}_{2}$ will decrease slightly in $l_{2}$ due to the impact with the diverter. Meaning that ${vc}_{2}>v_{1}$ where $v_{1}$ is the speed of the phone at $l_{2}.$

$$tdv=t_{2}-t_{1}=\frac{l_{2}}{{vc}_{2}}-\frac{l_{1}}{v_{1}}$$ (47)

The diverter must remain enabled ( d=1 ) during the period in which an S2 phone is located between the first and second limit ( $l_{1},l_{2}$ ) (Equation 48). An additional point to consider is that this operation must be carried out in such a way that the next incoming phone $S_{n+1}$ does not reach the first limit ${\;l}_{1}$ .

$$if\;\left(l_{1}{\leq S}_{n}\leq l_{2}\right)\;\&\&\;\left(S_{n+1}<l_{1}\right)\;\&\&\;\left(S_{n}==S2\right)\;\left\{\begin{array}{c} \;True\;d=1\;\\ False\;d=0 \end{array}\right.$$ (48)

##### 3.2.8.12 Constraint 12: binary variable constraint 3

For the screen extraction, a single type of disassembly is applied at once according to the state of the screen (S1 or S2) (Figure 13). However, to enable one type of disassembly to be selected while disabling the other, two variables x and y are employed, to be enabled or disabled (Equation 49).

x+y≤1 (49)

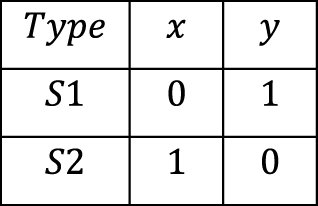




**FIGURE 13:**
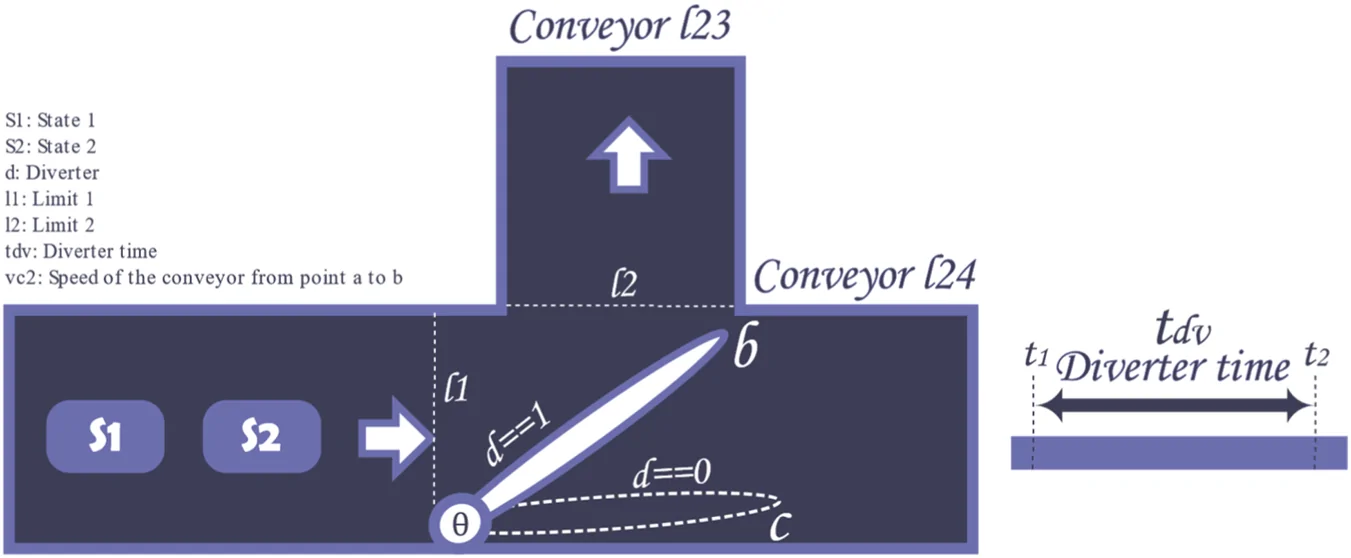
Illustration of constraint 12.

##### 3.2.8.13 Constraint 13: robot’s time constraint

Robot speed must be greater than conveyor speed to guarantee the efficiency of the robotic disassembly system (Figure 14). This relationship is mathematically based on the time required for an item to travel from point a to point b on the conveyor in which $d_{ab}$ is the distance between these points. Moreover, under scenarios where the robot needs to accelerate or decelerate, increasing speed enables smoother operation and better control of the robot’s movements. Allowing the robot to perform its tasks without causing disruption (Equation 50).

$$vr>{vc}_{1}\Leftrightarrow \frac{d}{t_{r}}>\frac{d_{ab}}{{tc}_{1}}$$ (50)

**FIGURE 14:**
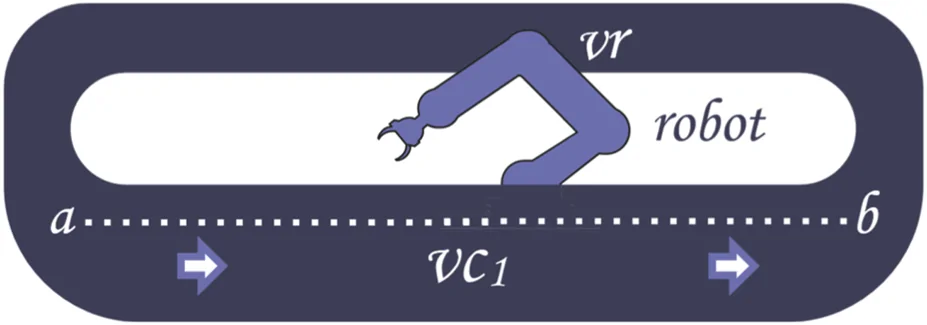
Illustration of constraint 13.

To improve the efficiency of the system, decreasing the time an item spends on the robot can significantly improve overall speed. As a result, the relation between these variables is given by the following formula in Equation 51:

$$t_{r}<\frac{{tc}_{1}.d}{d_{ab}}$$ (51)

##### 3.2.8.14 Constraint 14: diverter variable constraint 4

The diverter functions in two distinct states: enabled and disabled, respectively represented by d = 1 and d = 0. When the diverter is deactivated (d = 0), it is positioned so that items on the conveyor continue their path to the robotic disassembly in the event of a faulty screen $\left(S2\right)$ . In this state, the diverter is aligned to allow direct passage. However, when the diverter is activated (d = 1), it pivots to diverting the items from their path to an alternative one (Human disassembly). This is carried out when the screen is in proper condition $\left(S1\right)$ (Equation 52).

$$\left\{\begin{array}{c} d=1\;if\;\left(I_{1}=S1\right)\\ d=0\;if\;\left(I_{2}=S2\right) \end{array}\right.$$ (52)

##### 3.2.8.15 Constraint 15: heater constraint

When removing the screen from the phone, some screens are sealed by glue. Therefore, it is preferable to remove them using a heat gun (Figure 15). The heat gun’s operating temperature ht must be carefully controlled within a range. This range is derived based on the properties of the material, where ${ht}_{\min}$ (Equations 53, 54) is set slightly above the glue melting temperature to ensure efficient processing, and ${ht}_{\max}$ Equation 53, Equation 54 is set below the degradation temperature to avoid damaging the material. The reliability of the system is modelled to decrease or increase depending on a fluctuating temperature, emphasising the need to stay within the optimum range. To ensure the accuracy of the measurement, the temperature sensors and the control system must be calibrated periodically, where δ is the calibration error.

$${ht}_{\min}{<ht<ht}_{\max}$$ (53)

$$ht={ht}_{true}\pm \delta$$ (54)

**FIGURE 15:**
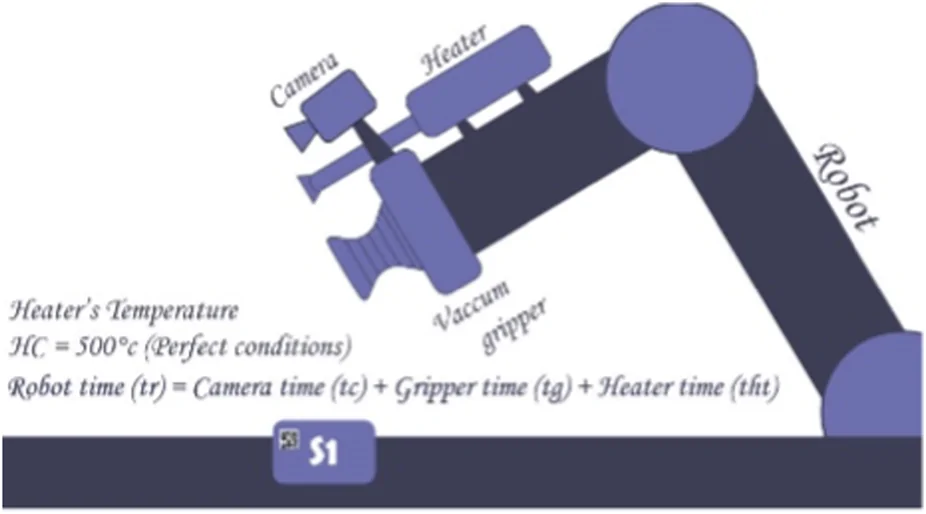
Illustration of constraint 15.

In the event of ht exceeding the range ( ${ht}_{\max}$ ), an alarm $\left(Alarm\right)$ is triggered, if it exceeds these limits for a sustained period of 30 s, the system shuts down. $t_{alarm}$ (Equation 55) is a timer which starts counting when it exceeds the interval and returns to 0 when it re-enters the range.

$$\left\{\begin{array}{l} t_{alarm}\;and\;Alarm=ON\;if\;\left({ht}_{\min}<ht{<ht}_{\max}\right)\\ t_{alarm}\;and\;Alarm=OFF\;otherwize\; \end{array}\right.$$

$$\left\{\begin{array}{l} Heater=ON\;if\;\left({ht<ht}_{\max}\;et\;t_{alarm}<30\;\sec\right)\\ Heater=OFF\;otherwize\; \end{array}\right.$$ (55)

##### 3.2.8.16 Constraint 16: binary variable constraint 5

Two binary variables x and y are introduced in the context of this station to represent specific operational conditions. The x variable is defined that x=1 if the phone does not need to be turned over and x=0 otherwise. In this way, the system can determine whether additional turning time is required during processing. In the same way the variable y is defined so that y=1 if the camera is present in the phone and y=0 otherwise. Such binary variables enable the system to adapt dynamically to varying configurations guaranteeing accurate and efficient timing under different conditions (Equation 56).

$$\left\{\begin{array}{l} x=1\;if\;the\;input\;require\;flipping\\ x=0\;otherwize\; \end{array}\right.$$

$$\left\{\begin{array}{l} y=1\;if\;the\;camera\;is\;present\;\\ y=0\;otherwize\; \end{array}\right.$$ (56)

##### 3.2.8.17 Constraint 17: flip-over unit constraint

An important requirement for the efficiency of the turning unit is that the time needed to turn the phone tf must be less than the time needed for the conveyor tcv to transport the phone from a to b (Figure 16). This constraint is crucial since it prevents the turning process from becoming a bottleneck in the system. Clearly, this constraint is crucial since it prevents the turning process from becoming a bottleneck in the system. Taking too long for the turnaround unit will delay the whole disassembly process, thus, creating a phone queue and increasing the overall processing time. Keeping tf (Equations 57, 58) below tcv allows us to maintain a smooth workflow. It guarantees that the tipping unit works in harmony with the conveyor promoting synchronization of operations and maintaining the desired throughput and enables the station to handle a greater number of telephones efficiently.

tf<tcv (57)

$$tf<\frac{d_{b}-d_{a}}{v_{cv}}$$ (58)

**FIGURE 16:**
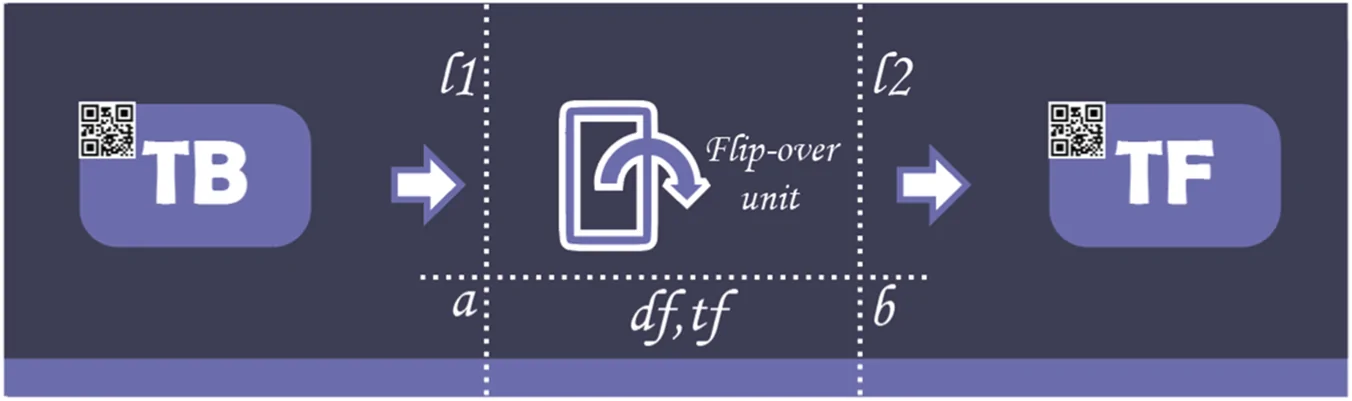
Illustration of constraint 17.

##### 3.2.8.18 Constraint 18: station’s inputs constraint

The Inputs $I_{1}$ and $I_{2}$ , are processed and classified into two types (Equation 59): Type Front ( TF ) and Type Back ( TB ). Ensuring a correct operation can be established by mathematical constraints that maintain the balance and flow of items through the station. The first is the flow balance constraint in which states that the total number of phones entering the station from the two inputs must be equal to the total number of items leaving the station Oe as $O_{TF}$ or ${\;O}_{TB}$ .

$$I_{1}+I_{2}=Oe\Leftrightarrow I_{1}+I_{2}=O_{TF}+O_{TB}$$ (59)

We also need to make sure that the number of TF and TB items coming from each input matches the phones that have been processed. This is done using type balance constraints in which the number of items of each type coming from the two inputs need to be equal to the total number of items of that type leaving the station. These constraints ensure the correct classification and balance of items as they pass through the station with the help of the camera tipping unit and robot (Equations 60, 61).

$$I_{1TF}+I_{2TF}=O_{TF}$$ (60)

$$I_{1TB}+I_{2TB}=O_{TB}$$ (61)

##### 3.2.8.19 Constraint 19: binary variable constraint 6

Two binary variables x and y , are introduced to represent specific operational conditions. We set the variable x so that x=1 if the phone has screws to be removed and x=0 otherwise. Using this, the system can determine whether additional unscrewing time is required during processing. In the same way that the variable y is defined so that y=1 indicates that the motherboard is present in the phone, and y=0 otherwise. Such binary variables enable the system to adapt dynamically to varying configurations, thereby guaranteeing a precise and efficient time under different conditions.

$$\left\{\begin{array}{l} x=1\;if\;the\;phone\;require\;unscrewing\;\\ x=0\;otherwize\; \end{array}\right.$$

$$\left\{\begin{array}{l} y=1\;if\;the\;motherboard\;is\;present\;\\ y=0\;otherwize\; \end{array}\right.$$

##### 3.2.8.20 Constraint 20: unscrewing time constraint

A time limitation is required to keep the total unscrewing time within an acceptable limit (Figure 17). This constraint is ensuring that the time required to unscrew any number of screws is less than the conveying time during this phase, especially when the number of screws to be unscrewed can be from 1 to 10. Maintaining this constraint is crucial to the smooth running of operations. Respecting this time constraint enables the process to remain efficient and avoid delays, whatever the variability of the number of screws (Equation 62).

$$ts<tcv\Leftrightarrow n.{ts}_{i}<tcv$$ (62)

$$\left\{i\in n|1\leq n\leq 10\right\}$$

**FIGURE 17:**
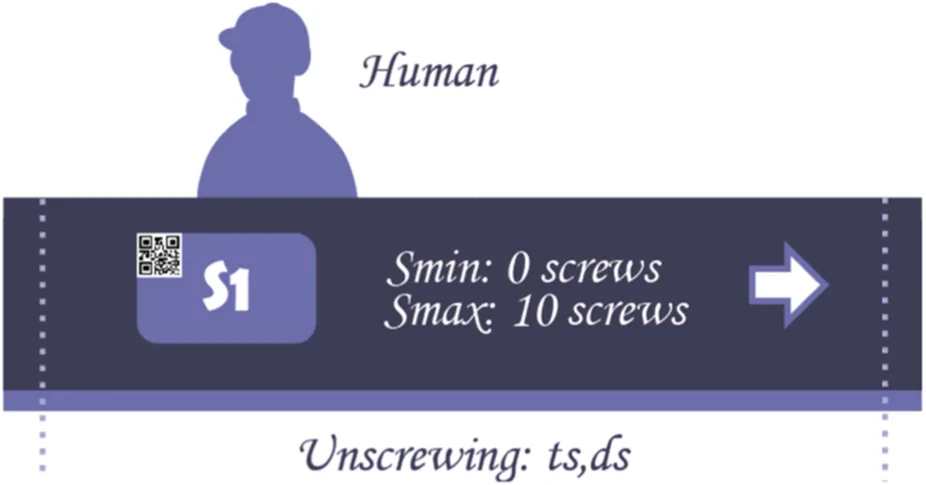
Illustration of constraint 20.

##### 3.2.8.21 Constraint 21: camera time constraint

A combined mathematical constraint is set to ensure that the extraction process te runs smoothly and with maximum performance (Figure 18). This constraint demands that the camera’s identification time is less than the robot’s extraction time also that the camera’s distance dc does not exceed a specified limit. When combining these constraints, it guarantees that the camera handles components quickly enough to keep up with the robot while components are transferred efficiently without excessive distance (Equation 63).

$$\left\{tc<tr\right\}\cap \left\{dc<b\right\}$$ (63)

**FIGURE 18:**
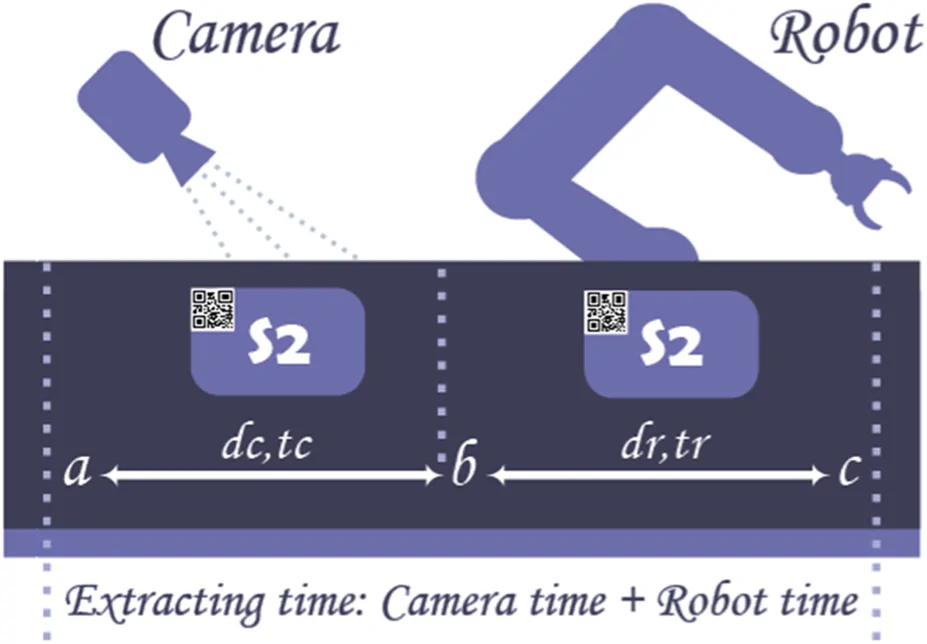
Illustration of constraint 21.

### 3.3 Collaborative implementation

Given that we work with both humans and robots, a collaborative approach must be proposed in this disassembly line leveraging both HRC and HRI frameworks optimizing time, error and increasing robots’ expertise across stations with both agents. We first need to select a station that contains both robots and humans while respecting variable thresholds and mathematical constraints, ensuring that tasks are executed effectively. Before simulations and Testing, we need to highlight that collaboration in improving this workflow (Figure 19).

**FIGURE 19:**
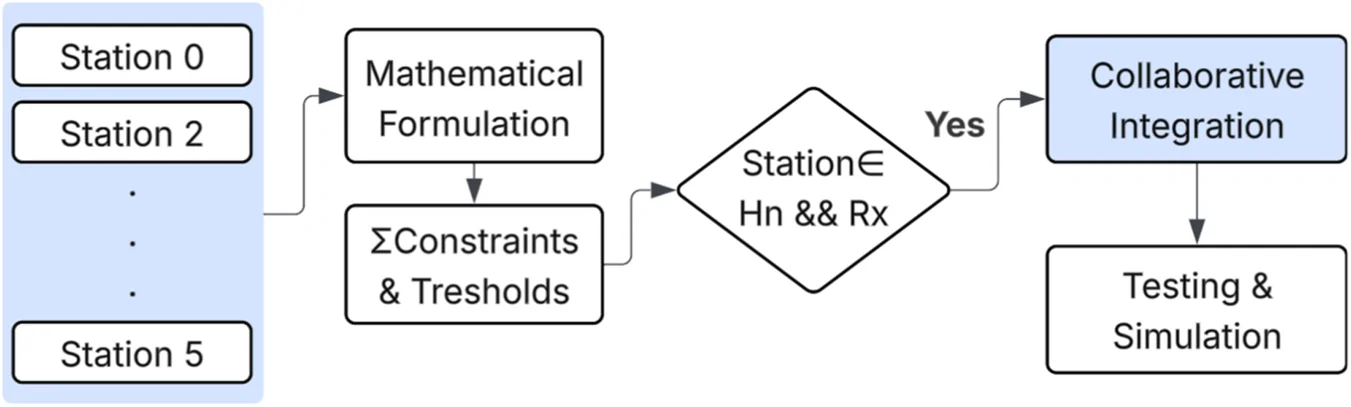
Collaborative implementation in station with both humans and robots.

Three stations in the disassembly line contain both humans and robots. Stations 0 and 3 focus on cooperation with humans and robots working independently but towards a common goal through hand signs or voice to exchange simple instructions and feedback. In Station 5, on the other hand, by contrast, a collaborative approach is adopted in which humans and robots actively share the extraction of the motherboard and depend on each other’s actions to complete complex operations. As a result, the human-robot combination of hand-signing and voice-signing is a highly effective tool. Such differentiation enables the system to adapt HRC strategies to the specific requirements of each station (Table 11).

**TABLE 11:** HRC approach at each station.

Stations	HRC approach	HRI
Station 0	Cooperation	Hand signs + voice
Station 3	Cooperation	
Station 5	Collaboration	

#### 3.3.1 Human robot interaction (HRI)

The HRI framework in this study is designed to seamlessly bridge communication between human operators and robotic systems, enabling highly adaptive disassembly processes (Wu et al., 2023). This multimodal interaction system leverages hand signs and voice commands, interpreted through a robust pre-trained recognition model. By integrating datasets of labeled hand gestures and voice commands into a centralized model. Cameras and microphones capture physical and verbal inputs, translating them into precise actions or comments for the robotic arm (UF LITE 6), show Figure 20. For example, a hand gesture signaling “good sorting” or “halt” is instantly recognized and executed by the robot.

**FIGURE 20:**
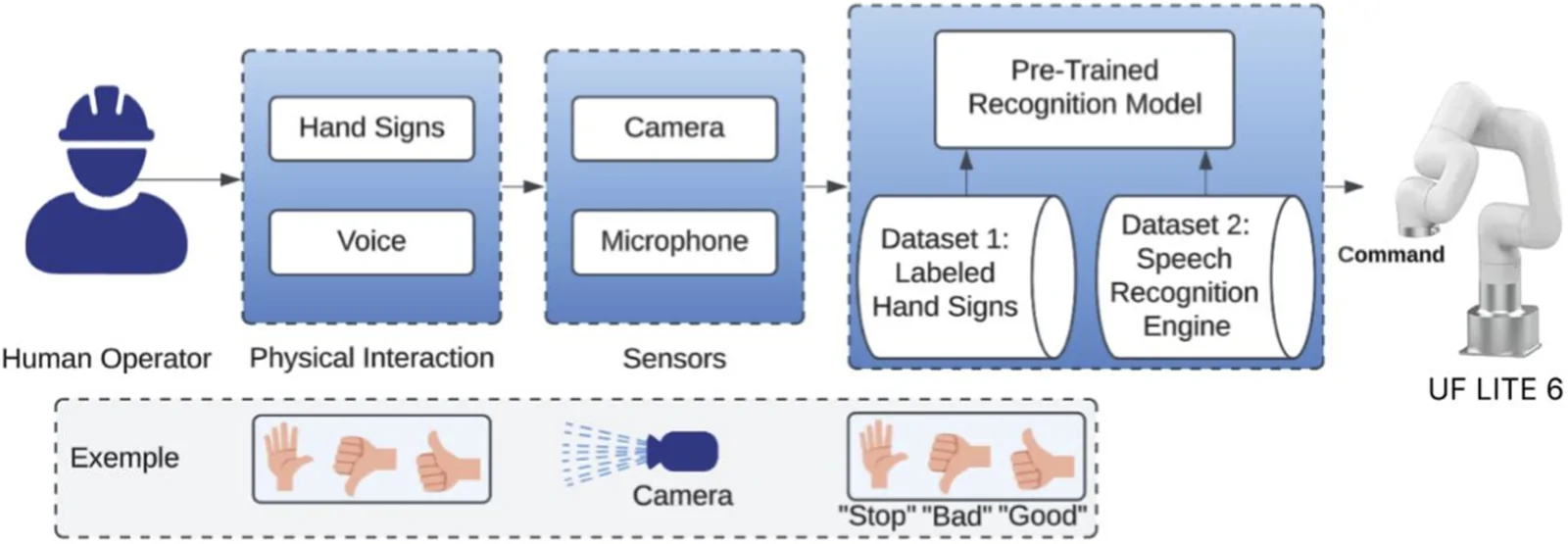
HRI interaction architecture.

One of the HRI communication protocols is a standardized taxonomy of nine distinct hand signs and corresponding voice commands to be used for a specific purpose (Figure 21; Table 12). Signs 1 and 2, or the “Good” and “Bad” voice commands respectively, provide sorting feedback with “thumbs up” or “Good” indicating successful sorting and “thumbs down” or “Bad” signaling sorting errors. Signs 3 and 4, or the “Stop” and “Continue” commands, are used as operational signals to interrupt or resume the disassembly process. Signs 5 and 6, or the “I take” and “You” commands work together to dynamically divide tasks between human operators and robots to ensure optimal collaboration according to task complexity. Meanwhile, signs 7 to 9, or the commands “Finish”, “Tool 1” and “Tool 2”, facilitate confirmation of task completion and tool-specific requests by ensuring uninterrupted workflows and dealing with dependencies between human and robotic systems. Such multimodal interaction ensures operator comfort and adaptability, as well as flexibility in the robotic disassembly process.

**FIGURE 21:**
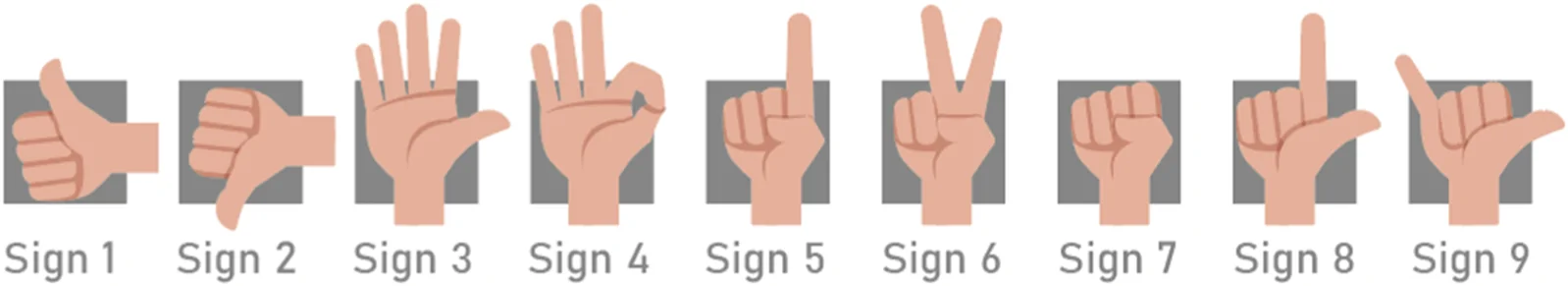
Proposed hand signs.

**TABLE 12:** Meaning of each hand sign and voice command.

Stations	Hand sign	Voice	Meaning
Station 0	Sign 1	“Good”	Good sorting
	Sign 2	“Bad”	Bad sorting
	Sign 3	“Stop”	Stop
	Sign 4	“Continue”	Continue
Station 3	Sign 5	“I Take”	Human takes the task
	Sign 6	“You Take”	Robot takes the task
	Sign 3	“Stop”	Stop
	Sign 4	“Continue”	Continue
Station 5	Sign 7	“Finished”	Human finish the task
	Sign 8	“Pick Tool 1”	Human needs tool 1
	Sign 9	“Place Tool 1”	Human finished using tool 1
	Sign 3	“Stop”	Stop
	Sign 4	“Continue”	Continue

The performance measures for four example voice commands “start,” “place tool 1” and “you take” or even other command such as “approach,” are shown in Figure 22. Three key measures are inference time (blue), real-time factor (red) and memory usage (green). All these commands have an inference time of between 2.3 and 2.8 s, which indicates a relatively constant processing speed. Memory consumption of the “Place tool 1” command is the highest here, probably due to its multi-word structure and linguistic complexity. The RTF values remain below 1 for all commands suggesting that the system is processing faster than real time in all cases. Real-time command has been done by Whisper-base (OpenAI, 2022), showing flexible recognition in robotic applications.

**FIGURE 22:**
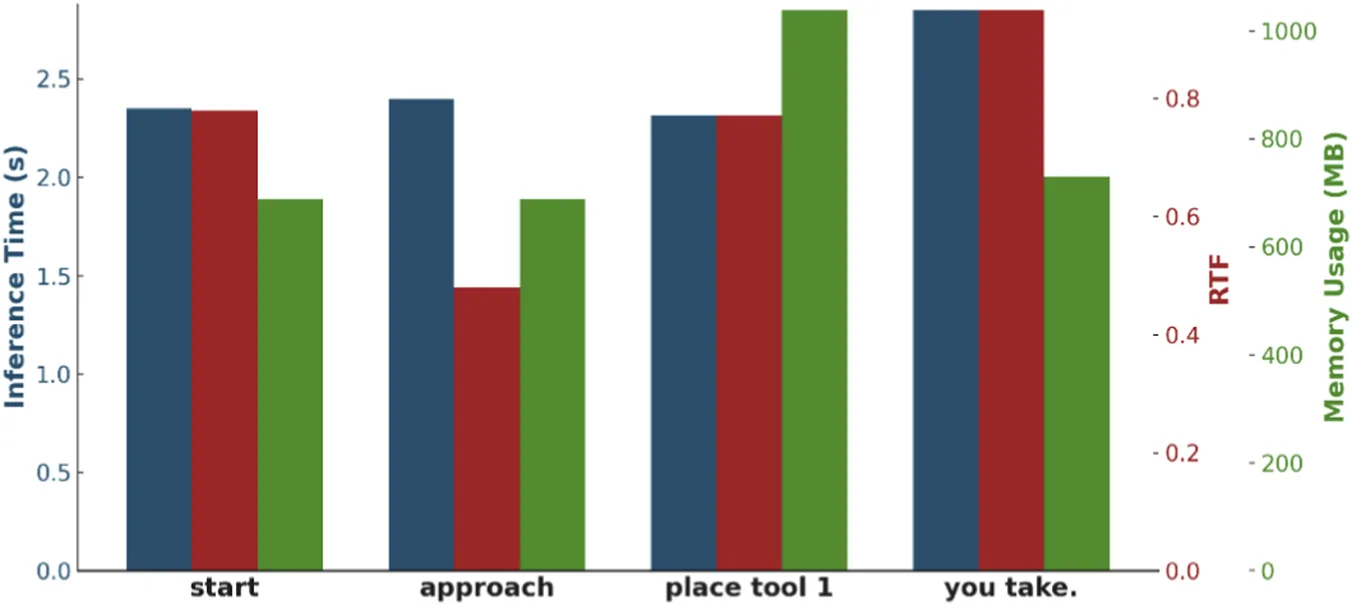
Performance of four voice commands using Whisper: inference time (blue), RTF (red), and memory usage (green).

The figure below shows the results of a hand gesture recognition system developed using the YOLOv12n model (Yang et al., 2025), trained on a custom dataset published on Roboflow Universe (Ameur, 2026). The dataset contains 317 annotated images distributed across four gesture classes (Sign1, Sign2, Sign3, and Sign4) and the trained model achieved a precision of 84.8% (Figure 23). The gestures were intentionally limited to four classes because these gestures were sufficient to represent the main interaction commands required in the proposed disassembly scenario. Three distinct gestures given as an example were accurately detected and classified in real time. In the first frame, “Sign2” is identified with a confidence of 0.91. The second frame correctly classifies “Sign3” with a confidence score of 0.94. And the third frame detects “Sign4” with a confidence of 0.91. The choice of YOLOv12n was motivated by its balance between high detection accuracy and low computational complexity since working on a raspberry pi 5.

**FIGURE 23:**
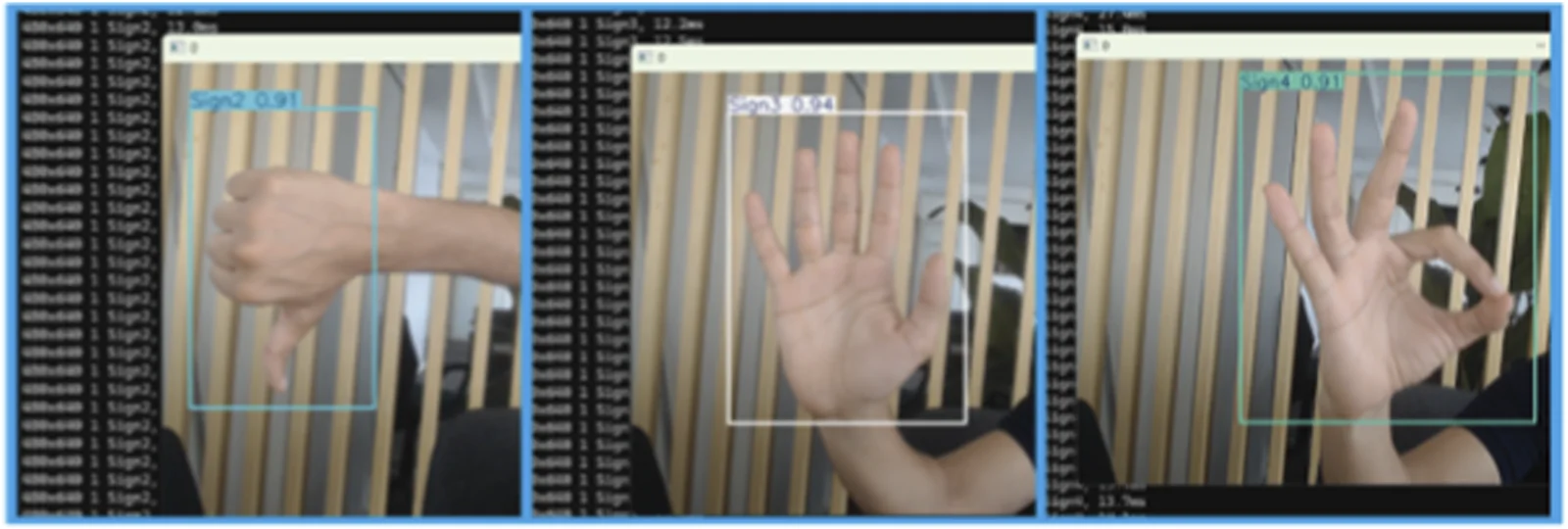
Hand signs detection using YOLOv12n.

#### 3.3.2 Constraints

In collaborative environments, constraints governing human-robot interactions are crucial to ensure a safe and adaptable collaboration. This subsection detail specific constraints integral to human-robot collaboration in station with both human and robot operators.

##### 3.3.2.1 Human cognitive overload constraints

Human operators must not be overburdened with complex or excessive tasks to prevent cognitive overload. The cognitive load $C_{h}$ at a station is calculated as the sum of the cognitive demand $c_{j}$ of each task j , scaled by the human performance coefficient $\phi_{h,}$ which accounts for factors such as expertise and fatigue:

$$C_{h}=\sum_{j=1}^{n_{h}}\;c_{j}\cdot \phi_{h}0$$

Here, $n_{h}$ represents the number of tasks assigned to the human. To prevent overload, the cognitive load must not exceed a predefined maximum threshold $C_{\max}$ :

$$C_{h}\leq C_{\max}$$

Cognitive workload is an important consideration in HRC disassembly, particularly when operators perform inspection, exception handling, and task-reassignment decisions. However, cognitive workload was not measured during the present study, and the task-demand coefficients and overload threshold were not calibrated using participant data.

##### 3.3.2.2 Proximity-aware interaction constraints

To maintain safety and efficiency in shared workspaces, the robot must operate within a defined proximity to the human. Let $D_{\text{actual},i}\left(t\right)$ denote the real-time distance between the human and robot at station i . The proximity must remain between the minimum safe distance $D_{\text{safe}}$ and the optimal working distance $D_{\text{optimal}}$ :

$$D_{safe}\leq D_{actual,i}\left(t\right)\leq D_{optimal}$$

The robot adjusts its speed $v_{r}\left(t\right)$ dynamically based on the proximity. The speed decreases as the robot approaches the human to ensure safety, modeled as:

$$v_{r}\left(t\right)=v_{\max}\cdot \left(1-\frac{\left|D_{optimal}-D_{actual,i}\left(t\right)\right|}{D_{optimal}}\right)$$

where $v_{\max}$ is the robot’s maximum speed. This ensures smooth, adaptive movements that maintain safety while optimizing collaboration.

##### 3.3.2.3 Feedback synchronization constraint

A successful transfer requires that the time ΔT , is defined as the interval between human interaction and robot feedback must not exceed a predefined maximum threshold $T_{\max}$ . The aim is to ensure a smooth coordination between man and robot during critical tasks such as extracting the motherboard at Station 5.

The human interaction time $T_{h}^{\text{interaction}}$ , is the moment when the human signals task completion, such as releasing the motherboard or providing a readiness signal. The robot feedback time $T_{r}^{feedback}$ , is the moment when the robot acknowledges the human’s signal and begins its subsequent task, such as aligning its gripper for the motherboard extraction. The time difference ΔT , is calculated as:

$$\Delta T=T_{r}^{feedback}-T_{h}^{\text{interaction}}$$

The constraint enforces that ΔT must not exceed $T_{\max}$ , the maximum allowable delay for effective synchronization:

$$\Delta T\leq T_{\max}$$

where $T_{\max}$ is a system-defined threshold, such as 2 s to ensure that the handover is completed promptly.

If $\Delta T>T_{\max}$ , an error correction process is initiated. The delay could arise from the human side ( $\tau_{h}$ ), such as a delayed readiness signal, or from the robot side ( $\tau_{r}$ ), such as issues with alignment or system readiness. The new synchronization time is recalculated as:

$$\Delta T_{\text{new}}=\Delta T+\tau_{\text{correction}}$$

where $\tau_{\text{correction}}$ is the additional time required to resolve the issue and ensure the system adapts to unexpected delays.

## 4 Testing and validation

This section presents a comprehensive evaluation of the proposed disassembly station through various simulation and integration scenarios. To ensure the feasibility and reliability of the system, we started by conducting a Robodk-based simulation first for digital modeling and conception for all the stations. Furthermore, a Real-Like simulation was applied to calculate times under real operational constraints. Followed by Constraint-Driven Python Simulation capabilities to test the process with random values to test error and calculate time. HRC Integration was done to assess adaptability in hybrid Stations. Finally, a comparative analysis summarizes the performance across the different configurations and benchmarks the results against existing disassembly approaches. As seen in Figure 24.

**FIGURE 24:**
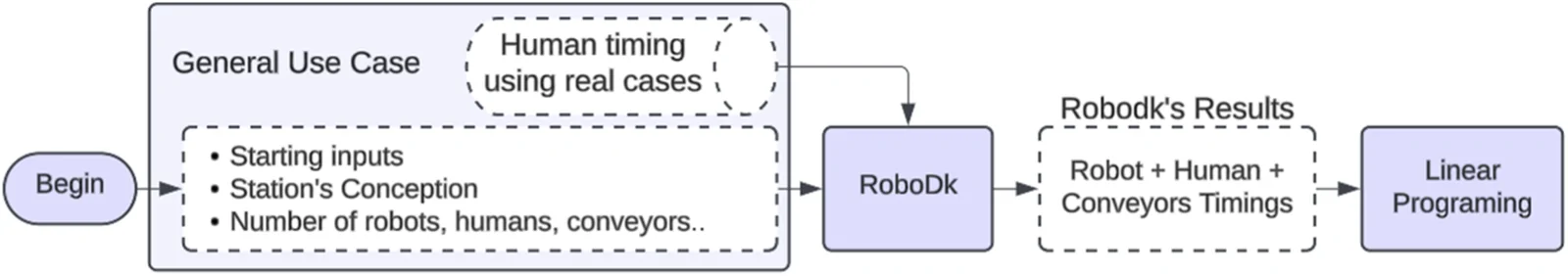
Suggested testing and validation.

### 4.1 Robodk simulation

The primary objective of RoboDK consists in modeling and visualizing the physical structure and interactions involved in the proposed disassembly process. This interface can be used to simulate the entire robotic and conveyor system. RoboDK simulation focuses on the modeling of the six interconnected stations to validate the layout and evaluate the various tasks and stations. The components of the end-of-life cell phones have been designed using CATIA V5 (Dassault Systèmes, 2025), capable of modeling complex geometries. Each component was individually and parametrically modeled to faithfully reflect real-world parts in formats compatible with the RoboDK environment (.stl), in which seamless integration into the simulation workflow Figure 25.

**FIGURE 25:**
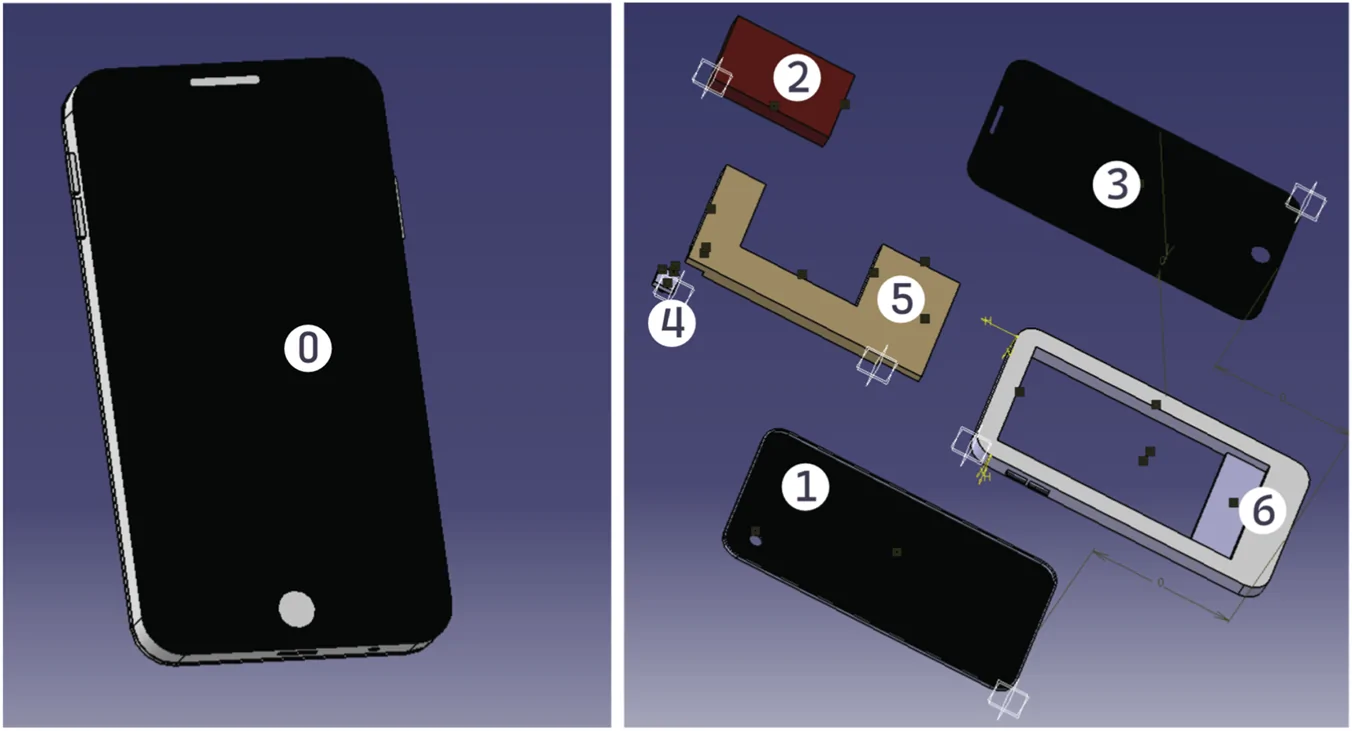
Designed EOL-phone input containing all 5 parts using CATIAv5.

The simulation was designed to match the proposed disassembly line as closely as possible (Figure 26), where efforts were made to reproduce the workflow, including tasks performed by robots, conveyors and other automated systems. Limitations of the RoboDK software posed problems for simulating human tasks that are essential for some disassembly operations. To tackle this problem, the next section of real experiments was carried out in which human operators performed the relevant tasks, helping us to incorporate realistic time estimates into the simulation. Certain machinery essential to the disassembly process, such as diverters and tilting mechanisms, are not available in the RoboDK simulation environment.

**FIGURE 26:**
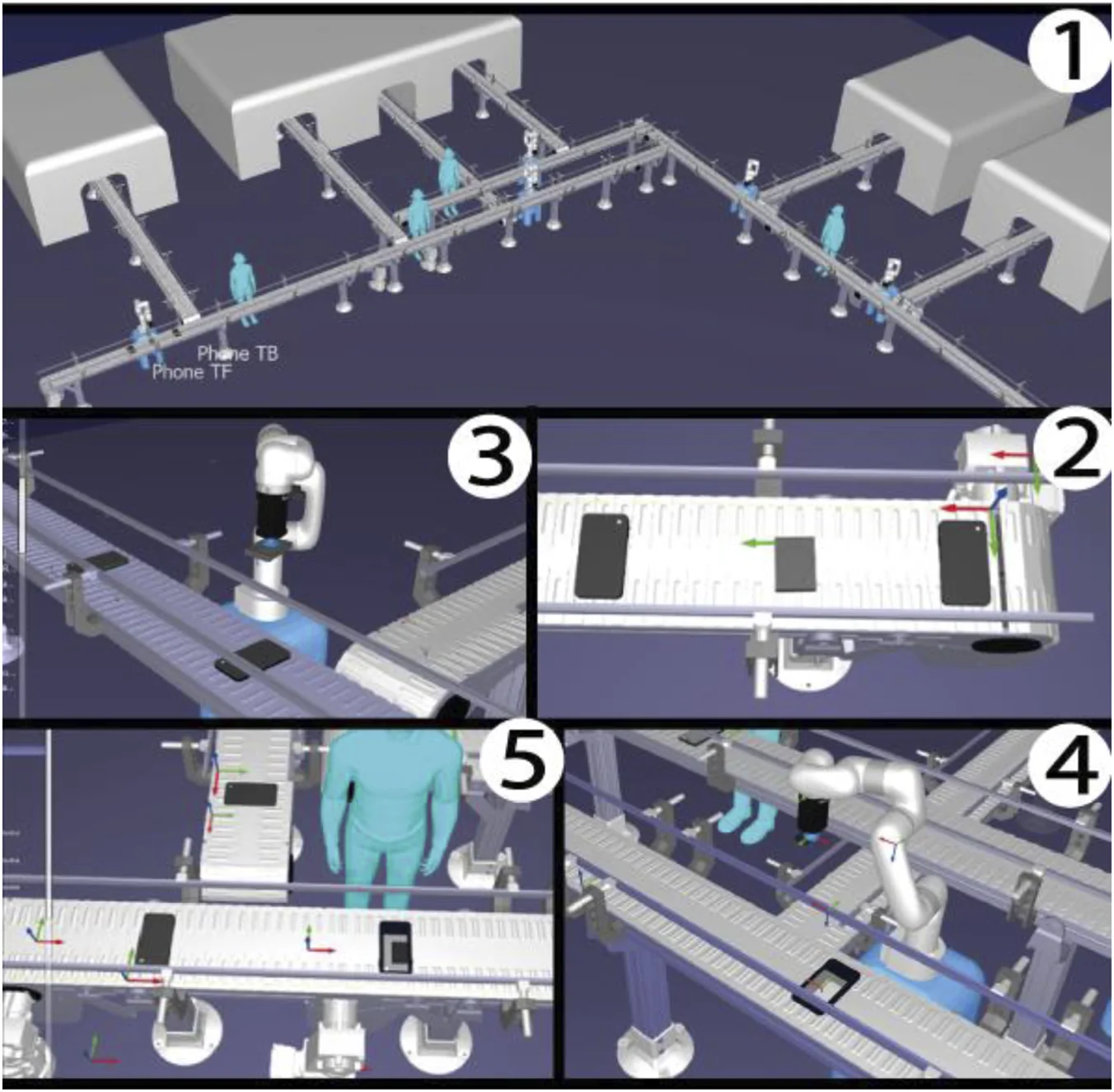
Process simulation using RoboDK.

Therefore, their functions were represented conceptually in the workflow but not physically modeled in the software (variable conv.). Our RoboDK simulation provided valuable information on the sequence of robotic tasks, conveyor movements and transitions between stations. The table shows the time required for each station based on the RoboDK simulation results, giving a total processing time of 2 min and 32.3 s. These results (Table 13) serve as a preliminary validation of the robotic tasks and highlight areas where further refinement is required to fully optimize the proposed system.

**TABLE 13:** Total time of all stations combined in RoboDK simulation (1 phone).

Station	Station 0			Station 1		Station 2	
Variable	R1	H1	Conv	H2	Conv	R2	Conv
Time (sec)	11.5	6.0	15.0	10.0	10.0	13.5	10.0
Station	Station 3			Station 4		Station 5	
Variable	H3	R3	Conv	R4	Conv	R5	Conv
Time (sec)	3.0	12.6	15.0	11.0	12.0	10.7	12.0
Total Time: 2 m 32.3 s (0 constraint)							

### 4.2 Single-station test

To approximate realistic operating conditions, a real-like simulation was performed using U factory 6 lite and roads were demonstrated as conveyors. As illustrated in Figure 27, we simulated Station 0 where the process begins with the inputs placed on the conveyor. The object is detected at Ts2, followed by robotic manipulation steps at Ts3 = 2.2 s, followed in Ts4 = 3.1 s by putting the unwanted input at the recycling line and final positioning at Ts5 = 4 s. This setup provides a controlled yet dynamic environment that supports the precise measurement of execution times under all predefined thresholds and operational constraints to enable accurate validation of system performance and timing efficiency. The value ranges of the main simulation variables and operational parameters are summarized in Table 14.

**FIGURE 27:**
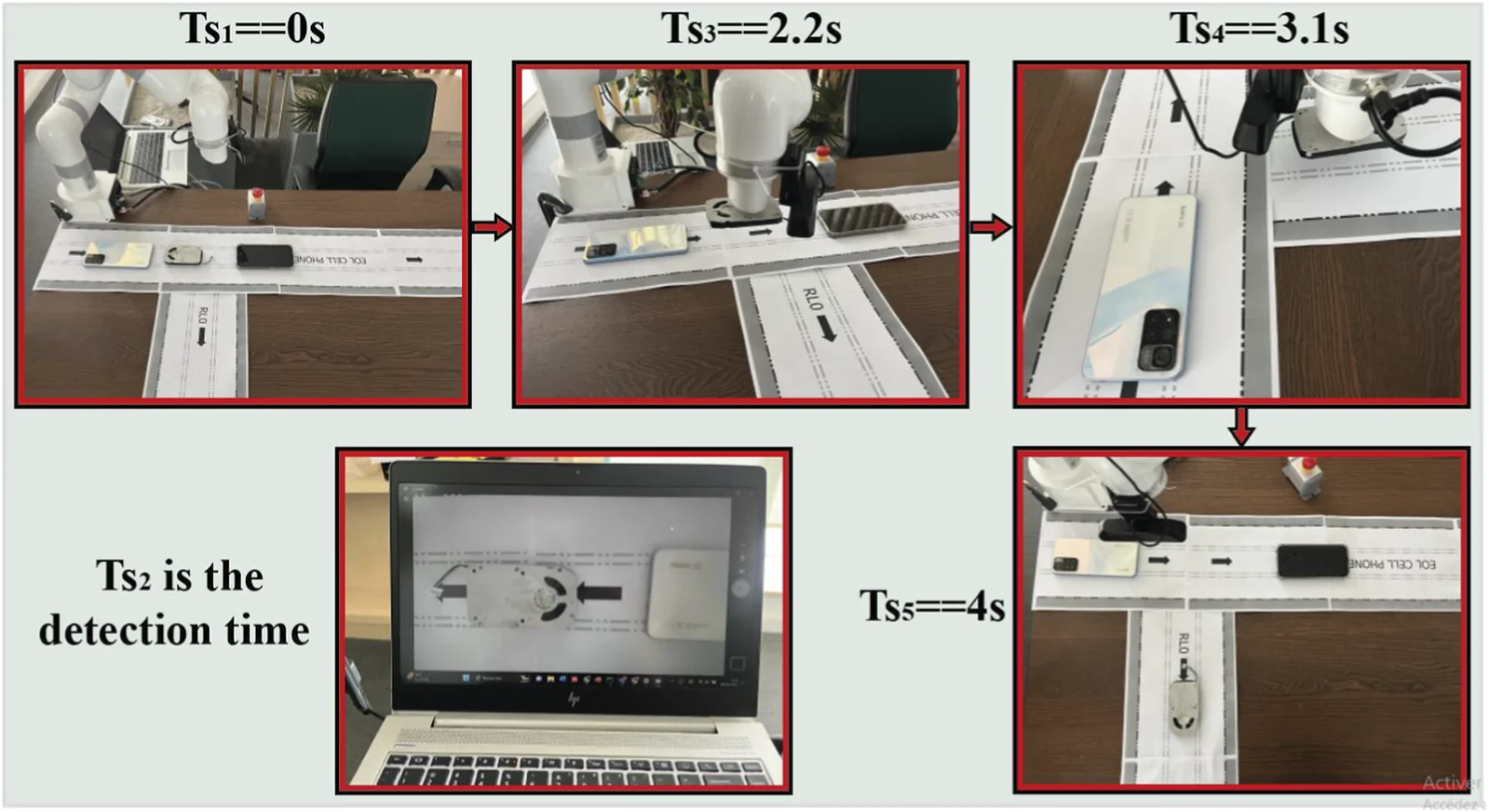
Single-station UF Lite 6 bench test and timing measurements.

**TABLE 14:** Value variation of each variable.

Variable	Name	Value variation	Reference
${tr}_{i}$	Robot’s time	$10s\leq {tr}_{i}\leq 15s$	Our model (IV.2. real-like simulation)
${th}_{i}$	Human’s time	$5s\leq {th}_{i}\leq 12s$	
${tc}_{ij}/L_{i}$	Conveyor’s time	$10s\leq {tc}_{ij}/L_{i}\leq 20s$	
$D_{i}$	Distance	$0.1m\leq D_{i}\leq 5m$	
$D_{safe}$	Safe distance	$0.2m\leq D_{i}$	
${vc}_{i}$	Conveyor’s speed	$0.1m/s\leq {vc}_{i}\leq 1.5m/s$	
ts	Unscrewing time	8s≤ts≤30s	
te	Extraction time	5s≤te≤10s	
ht	Heater time	10s≤ht≤30s	
$R_{u}$	Unrecognized input	Binary	
$t_{f}$	Flip over time	1s≤tf≤3s	
$K_{i}$	Coefficient	0.1≤Ki≤1.0	
${tdv}_{i}$	Diverter’s time	$3s\leq {tdv}_{i}\leq 10s$	
I / D / L / R	Camera’s influence	1≤I / D / L / R≤10	
$P\;/\;W\left(d_{cog}\right)\;/\;\tau_{i}$	Robot’s constraints	Mentioned in constraint part	
tq	RFID’s time	1s≤tq≤5s	
td	Decision time	1s≤td≤10s	

### 4.3 Constraint-driven python simulation

Now, while RoboDK provides a visualization of the disassembly line and the Single-Station UF Lite 6 Bench Test enables precise threshold calculations, the Python-based simulation serves as a rigorous test environment to ensure that every component of the line operates correctly under a variety of conditions. Within this simulation, all the critical constraints such as robot accessibility, payload limits, task priority and conveyor functionality have been explicitly modeled. Using a Python-based approach in which we were able to carry out detailed validation of the entire system by confirming its ability to handle realistic variations. In addition, by testing the line under a wide range of constraints, the Python simulation ensured that the disassembly process could operate reliably without exceeding operational limits. In this table, the key variables and their variations used in the disassembly process simulation are categorized as operational parameters. References indicate data sources from RoboDK and experimental logs to validate constraints and inputs.

An average processing time was calculated for the simultaneous disassembly of 10 phones over 200 iterations (Figure 28). This approach enabled us to obtain a reliable average that takes into account variations in the disassembly process, ensuring consistent results and minimizing the impact of outliers. Analyzing data from several iterations, we made sure that the calculated average accurately reflects the system’s operating efficiency under the given conditions.

**FIGURE 28:**
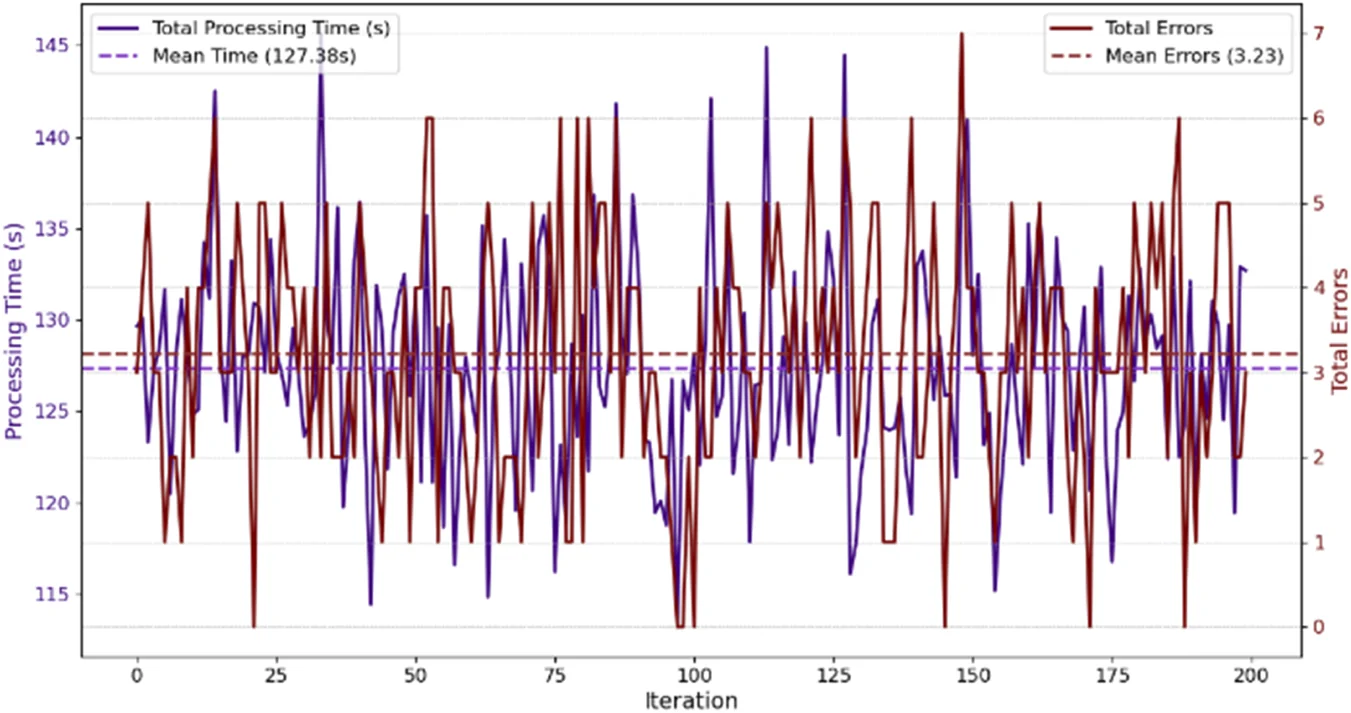
Processing time and error of 10 phones over 200 iterations.

The results of the disassembly process of 10 phones in six stations reveal significant variations in processing times and consistently high error rates. An error is recorded when a station output differs from the predefined state of the phone and requires correction or repetition. For each station, 10 phones were simulated over 200 iterations, resulting in 2,000 trials. The error rate was calculated as the number of failed trials divided by 2,000. Station-specific errors include incorrect classification, routing, detection, extraction, flipping, diverter operation, and unscrewing. These errors were programmatically in the Python simulation using predefined station-specific probabilities and random sampling. A total processing time of 955.37 s was recorded, with Station 5 being the longest (177.80 s), as summarized in Table 15. The shortest time was recorded by station 0 (127.38 s) reflecting relatively high efficiency. All stations had particularly high error percentages ranging from 3.2% to 4.1% with Station 5 having the highest error rate.

**TABLE 15:** Total time and error of each station.

Station 0		Station 1		Station 2	
Time (sec)	Error	Time (sec)	Error (%)	Time (sec)	Error (%)
127.38	3.23	160.75	3.5	168.30	3.7
Station 3		Station 4		Station 5	
Time (sec)	Error	Time (sec)	Error (%)	Time (sec)	Error (%)
163.34	4.0	157.80	3.8	177.80	4.1
Total Time: 955.37 s					

The RoboDK result (152.3 s) represents a nominal single-phone sequence, while the Python result (955.37 s) corresponds to a simulated 10-phone batch under varying parameters. Therefore, 95.54 s/phone is a simulation-based average, not a measured industrial throughput.

### 4.4 HRC integration

The section highlights the benefits of HRC in hybrid disassembly stations in which humans and robots jointly perform tasks (station 0-3-5). Integrating HRI using voice commands will allow dynamic reassignment of tasks, rapid error recovery, and also as a significant reduction in downtime. The scenarios demonstrated below will show how HRC improves responsiveness under real-life conditions. In Station 0, Let’s imagine that the robot mistakenly classifies an EoL phone as an unwanted item. If there were no HRC, the human need to wait for the robot to complete its task before intervening as this would cause delays. Using voice interaction the operator can simply say “bad,” prompting the robot to immediately reprocess the item. This reduces the correction cycle while activating simultaneous tasks to be managed, as shown by the reduction in idle time in the HRC timeline (Figure 29).

**FIGURE 29:**
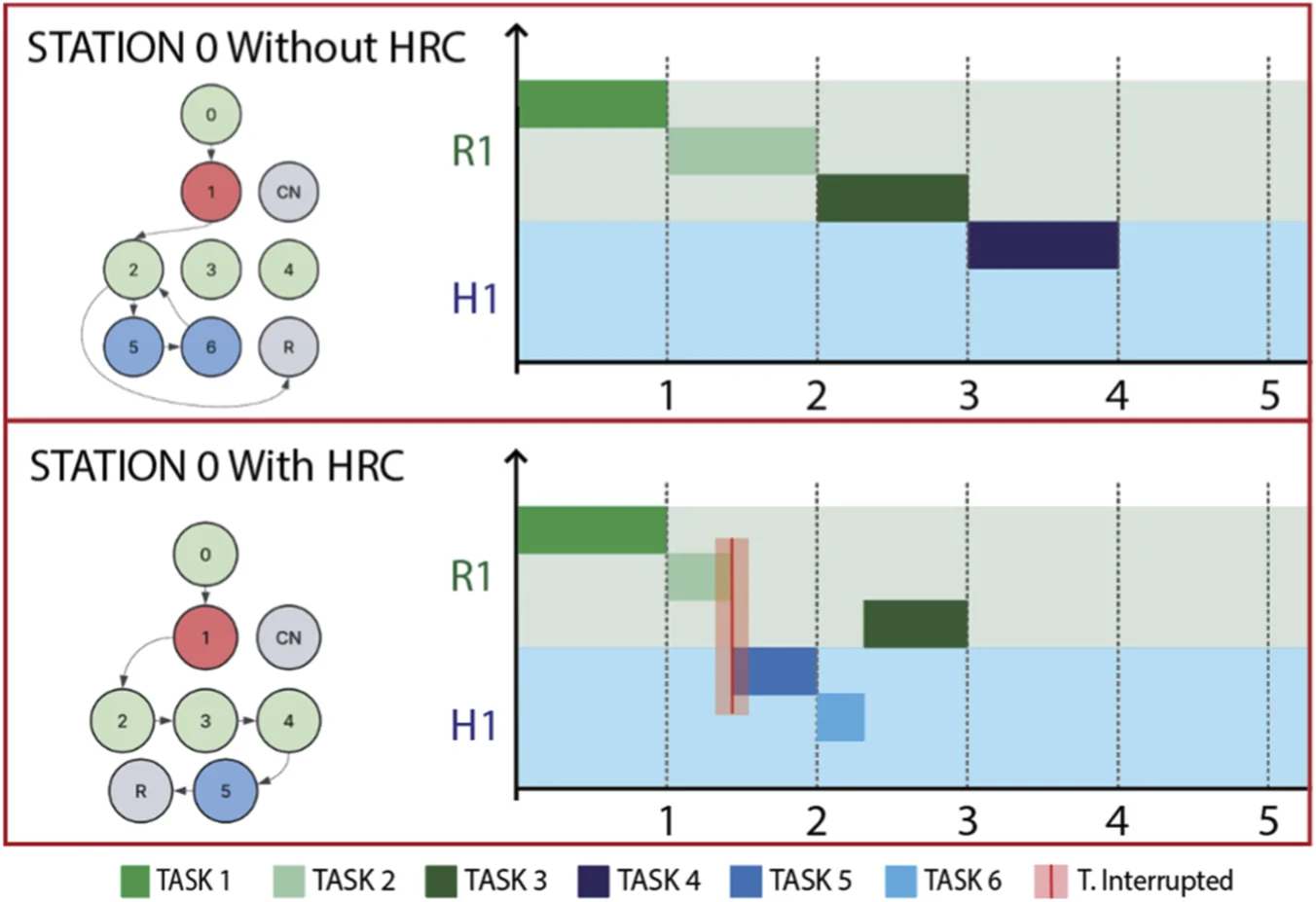
Gantt chart of scenario 1.

In Station 3, the division of tasks here is based on the condition of the screen involved where the human disassembles intact screens while the robot deals with damaged ones. If sorting fails and the human receives a broken screen, the device usually must be rerouted. With the HRC system, the operator can give the command “you take,” making the robot to take over the task on the spot. This eliminates queuing and optimizes workflow continuity (Figure 30).

**FIGURE 30:**
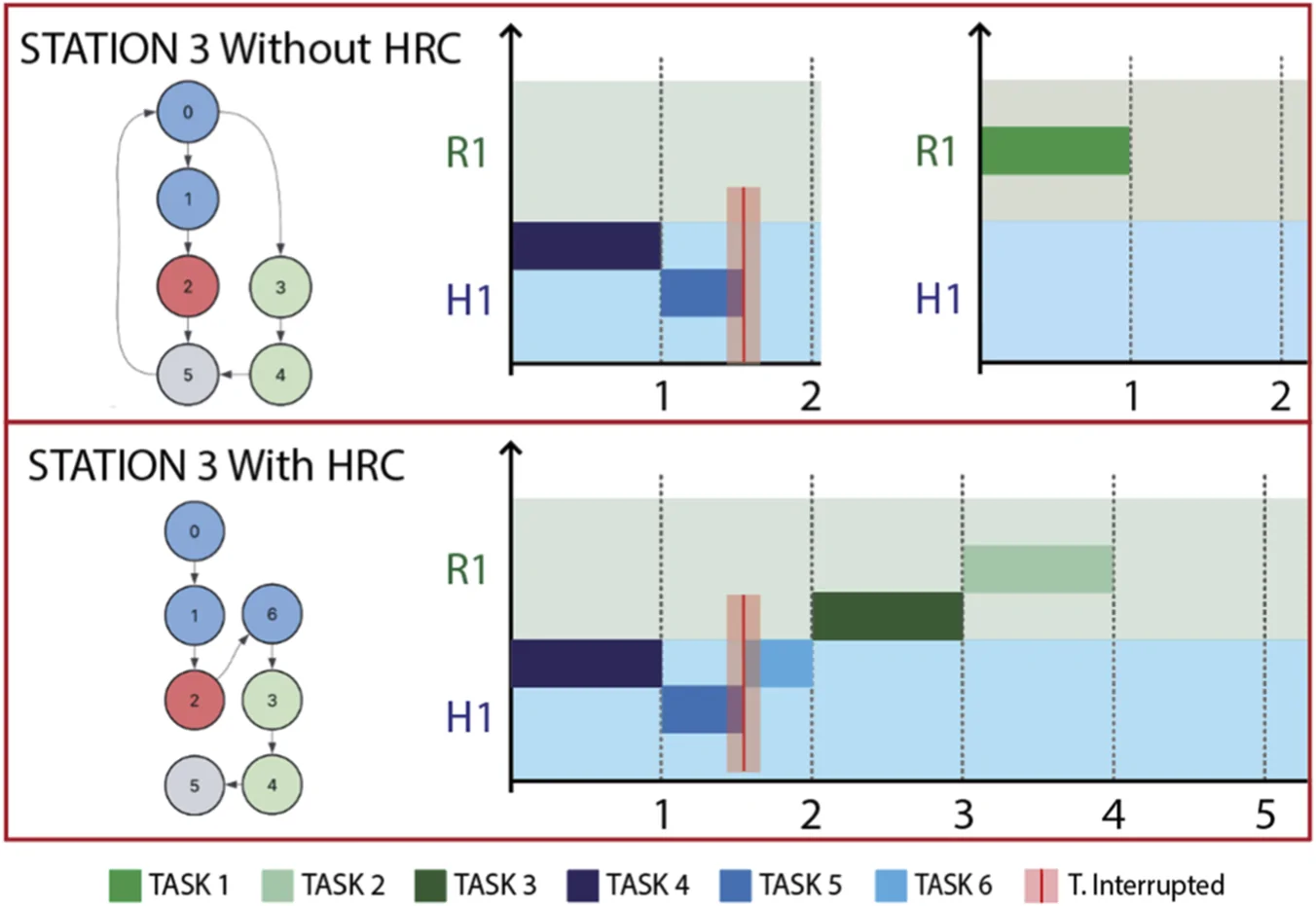
Gantt chart of scenario 2.

In this station 5, the human is responsible for unscrewing components before robotic extraction. If the human steps away in a sudden emergency or break, the robot is forced to wait. Using HRC, the human can say “continue” allowing the robot to complete both tasks autonomously. This avoids a standstill time and maintains operational flow, as reflected in the uninterrupted execution timeline (Figure 31).

**FIGURE 31:**
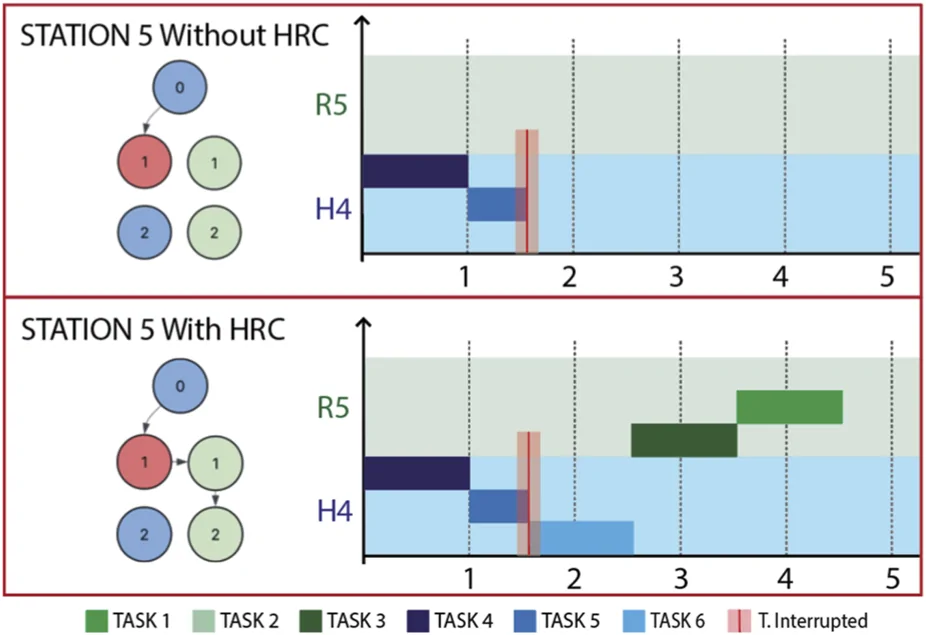
Gantt chart of scenario 3.

### 4.5 Comparative table

The system proposed a solution with flexibility and collaboration, in addition to process transparency, as shown in Table 16. Unlike fully automated systems such as Apple’s Liam or Daisy that are optimized exclusively for iPhones and rely heavily on rigid mechanical separation, our system supports different cell phone models and integrates robotic precision with human cognitive flexibility. Unlike previous collaborative setups such as those by Ambrosch et al. and Pamninger et al. using voice-activated HRC, we offer real-time error correction, adaptive task allocation and reduced downtime costs. In contrast, systems such as Liam and Daisy achieve higher throughput through dedicated automation for specific iPhone models. While the 95.54-s disassembly speed per phone may seem slower than systems designed for single devices, it reflects a more generalized, adaptable and accurate process across heterogeneous EoL phones, along with improved recovery rates and minimal error propagation.

**TABLE 16:** Comparative table with similar approaches.

System	Collaboration type	Targeted devices	Disassembly method	Processing time	Error rate
Proposed Process	Collaborative (robot + human)	Various mobile phones	Mechanical separation, Heating, Unscrewing.	95.54 s per phone	3.23% to 4.1%
Liam 1.0 (Rujanavech et al., 2016)	Fully automated	iPhone 5s	Mechanical separation	∼11 s per iPhone	Not mentioned
Liam 2.0 (Heffernan, 2024)	Fully automated	iPhone 6s	Mechanical separation	∼8–10 s per iPhone	Not mentioned
Daisy (Young, 2018)	Fully automated	iPhones	Mechanical separation	∼200 iPhones/hour	Not mentioned
Kopacek and Kopacek (2006)	Semi-automated	Various mobile phones	Drilling, milling, suction grippers for PCB removal	Not mentioned	Not mentioned
He et al. (2020)	Fully automated	Various smartphone circuit boards (Huawei, Xiaomi, Apple)	Visual inspection, heating, CPU transfer robot	36 s (first stage), 21 s (second stage)	∼12% (CPU extraction success 88%)
Pamminger et al. (2019)	Collaborative (robot + human)	Various mobile phones	Clamping, heating, unscrewing modules	∼145 s per phone (varies)	Not mentioned
Ueda et al. (2024)	Fully automated	Various mobile phones	Freezing, X-ray scanning, screw disabling, impact disassembly	∼6 s per phone	11.7% (success rate 88.3%)

The proposed framework was evaluated at three complementary levels: a digital simulation of the six-station architecture using RoboDK, a single-station bench test using the UF Lite 6 cobot, and a Python-based simulation performed over 200 iterations. The RoboDK model evaluates the system layout and task sequence, the bench test validates selected robotic operations, and the Python simulation estimates processing time and error rates under varying parameters. The proposed system is scalable because its modular stations and constraint-driven control can accommodate additional phone models, tasks, and robots without redesigning the full line. Voice commands enable rapid task reassignment and error correction, reducing downtime. Its sustainability comes from selective component recovery, reduced damage, and improved handling of heterogeneous e-waste, although industrial-scale validation is still required.

### 4.6 Limitations

The present study does not establish the economic profitability of the proposed system. A complete industrial assessment would require the purchase and integration costs of robots, conveyors, cameras, safety devices, maintenance, energy consumption, operator wages, recovered-component value, and cost per processed phone. Although labor cost is a known limitation of manual disassembly, the proposed architecture does not assign all operations to humans. Instead, human involvement is limited to tasks where flexibility, visual judgement, or delicate manipulation may avoid component damage and costly system stoppages. Economic viability may therefore depend on whether the additional labor cost is compensated by reduced changeover requirements, improved recovery of reusable components, and the ability to process mixed phone models. These benefits were not monetized in the current simulation and require validation through a pilot-scale cost analysis. In addition, the safety constraints were evaluated conceptually and in simulation; complete industrial deployment would require physical validation, risk assessment, protective separation monitoring, emergency-stop testing, and compliance with applicable collaborative-robot safety standards. A direct comparison of flow errors with existing systems was not possible because most studies do not report this metric or use equivalent error definitions. Therefore, comparisons were limited to the available error or success rates, and standardized benchmarking remains necessary in future work. A further limitation is voice-command latency which is expected when running Whisper-base that can be unsuitable for time-critical robot commands.

## 5 Conclusion and perspectives

This study presents a comprehensive design and validation of an HRC disassembly station for EoL phones that addresses the critical challenges posed by growing volumes of e-waste and the need for more sustainable recycling practices. The system integrates HRC and HRI using voice and intuitive gesture commands that allow for dynamic task reassignment, real-time error correction, and enhanced safety measures, providing a clear departure from rigid, single-device-centric approaches such as those implemented by Apple’s proprietary systems. Using Single-Station UF Lite 6 Bench Test and RoboDK modeling, the new architecture leverages Constraint-Driven Simulation with over 30 operational and collaborative constraints in mind for a highly flexible and adaptable solution for recycling operations. In terms of the future, there are several opportunities to further improve the performance and applicability of this system. For example, reducing disassembly time and error rates have a key for increasing performance and reliability while incorporating advanced trajectory planning and decision-making frameworks like reinforcement learning algorithms should play an important role in optimizing robotic movements and dynamic task reallocation. Additionally, the extension of the system’s sensory capabilities through multimodal data acquisition encompassing 3D vision and tactile feedback should improve precision in the manipulation of various device and material architectures. Technical improvements aside, a broader perspective involves adapting this system to disassemble other types of electronic waste, such as tablets, portable devices, and consumer electronics and so extend its impact and align with the principles of the circular economy. Future work will also conduct participant-based ergonomic and cognitive-load experiments to calibrate the proposed workload model and compare fixed task allocation with workload-aware reassignment in terms of cycle time, error rate, fatigue, and perceived workload. Deploying these collaborative disassembly systems on an industrial scale will have to require overcoming the challenges of economic viability and integration into existing recycling infrastructures. Yet the results of this study and the methodologies has proposed highlight the potential of constraint-driven, voice-assisted collaborative disassembly systems to meet the requirements of next-generation e-waste recycling efforts, thus fostering a more resource-efficient and sustainable future.

## Data Availability

The original contributions presented in the study are included in the article/supplementary material, further inquiries can be directed to the corresponding author.
